# Cerebellar Nuclear Neurons Use Time and Rate Coding to Transmit Purkinje Neuron Pauses

**DOI:** 10.1371/journal.pcbi.1004641

**Published:** 2015-12-02

**Authors:** Shyam Kumar Sudhakar, Benjamin Torben-Nielsen, Erik De Schutter

**Affiliations:** 1 Computational Neuroscience Unit, Okinawa Institute of Science and Technology, Onna-son, Okinawa, Japan; 2 Laboratory of Theoretical Neurobiology and Neuro-engineering, University of Antwerp, Wilrijk, Belgium; 3 Biocomputation Research Group, University of Hertfordshire, Hertfordshire, United Kingdom; The Krasnow Institute for Advanced Studies, UNITED STATES

## Abstract

Neurons of the cerebellar nuclei convey the final output of the cerebellum to their targets in various parts of the brain. Within the cerebellum their direct upstream connections originate from inhibitory Purkinje neurons. Purkinje neurons have a complex firing pattern of regular spikes interrupted by intermittent pauses of variable length. How can the cerebellar nucleus process this complex input pattern? In this modeling study, we investigate different forms of Purkinje neuron simple spike pause synchrony and its influence on candidate coding strategies in the cerebellar nuclei. That is, we investigate how different alignments of synchronous pauses in synthetic Purkinje neuron spike trains affect either time-locking or rate-changes in the downstream nuclei. We find that Purkinje neuron synchrony is mainly represented by changes in the firing rate of cerebellar nuclei neurons. Pause beginning synchronization produced a unique effect on nuclei neuron firing, while the effect of pause ending and pause overlapping synchronization could not be distinguished from each other. Pause beginning synchronization produced better time-locking of nuclear neurons for short length pauses. We also characterize the effect of pause length and spike jitter on the nuclear neuron firing. Additionally, we find that the rate of rebound responses in nuclear neurons after a synchronous pause is controlled by the firing rate of Purkinje neurons preceding it.

## Author Summary

Neurons can transmit information by two different coding strategies: Rate coding, where the firing rate of the neuron is vital, and time coding where timing of individual spikes carries relevant information. In this study we analyze the importance of brief cessations in firing of the presynaptic neuron (pauses) on the spiking of the postsynaptic neuron. We perform this analysis on the inhibitory synaptic connection between Purkinje neurons (presynaptic) and nuclear neurons (postsynaptic) of the cerebellum. We employ a computational model of nuclear neurons and “synthetic” Purkinje neuron spike trains to study the effect of synchronous pauses on the spiking responses of nuclear neurons. We find that synchronous pauses can cause both well-timed spikes and increased firing rate in the nuclear neuron. In addition, we characterize the effect of pause length, amount and type of pause synchrony, and spike jitter. As such, we conclude that nuclear cells use both rate and time coding to relay upstream spiking information.

## Introduction

Cerebellar nucleus (CN) neurons are crucial to the olivo-cerebellar circuit as they provide the sole output of the entire cerebellum [1,2]. CN neuron’s firing patterns are of great importance for motor related tasks and representation of movement parameters [3]. Within the cerebellum, their direct upstream connections originate from the Purkinje neurons (PNs). PN firing patterns are vital for CN neuron’s functioning, as CN neurons receive strong inhibition from many PNs [4,5] combined with modest depression of the synapse through spillover of GABA from many release sites [6].

PNs exhibit an elaborate firing pattern characterized by simple spikes and complex spikes [7]. Simple spikes are driven by spontaneous intrinsic firing [8,9] but are also modulated by excitatory input through the ascending axons and parallel fiber synapses [10] and by feed-forward inhibition through the granule neuron-interneuron-PN pathway [11]. The combination of intrinsic firing and synaptic input results in highly regular spikes with typical short pauses [12,13], where a pause is a short cease in firing. Given this elaborate firing pattern in PNs, how can the downstream CN neuron make sense out of this signal?

Several coding strategies have been proposed for the CN neurons. Broadly, these strategies can be categorized as rate coding or time coding. For a long time it was assumed that CN neurons receive information from PNs by means of a rate code. In cats, CN neurons (recorded from the anterior interpositus nucleus) exhibited rate modulations during locomotion [14]. Simultaneous paired recordings from Purkinje and CN neurons show that the modulation responses of the pair is not always reciprocal, which implies that firing modulation characteristics of CN neurons reflect combined activity of many of its presynaptic PNs and mossy fiber input [15]. More recently, time coding was proposed by Person & Raman and entailed time-locking to synchronous input from the PNs [5]. The rationale is that synchrony of a small fraction of PN inputs cause brief periods of relief from inhibition, which makes the CN neuron’s spiking time-locked to the synchronous input.

Systematic analysis of simple spikes from neighboring PNs in anaesthetized rats revealed that spikes associated with pauses in firing are far more synchronized (±2 ms precision) than regular firing simple spikes [7], which indicates a role for pause synchrony in precise timing. The study also reports that, in pairs of PNs, approximately 35% spikes were precisely synchronized and 13% of pauses were synchronized either by their beginning spikes or ending spikes. Moreover, cross-correlograms of neighboring PNs calculated only with pause beginning spikes or ending spikes exhibited sharp peaks and the correlations thus obtained were not distinguishable from each other. So it seems that synchronization of both pause beginning and pause ending spikes are equally probable in neighboring rat PNs. In another study (also anaesthetized rats), timing of pause beginning spikes (and also ending spikes) was significantly correlated (±5 ms) in pairs of PNs which exhibited high complex spike synchrony [16]. Coupled pairs of PNs were likely to pause together (±5 ms precision) with an increased probability of 82% and end their pauses together with an increased probability of 39%. In a follow-up review, De Schutter and Steuber proposed that PN simple spike trains combine both rate and temporal codes [17]. The pauses in simple spikes act as a temporal code that may cause a well timed rebound firing of CN neurons when the pauses are synchronized across a population. At the same time, the regular spikes act as a rate code that determines the magnitude of succeeding rebound responses. So it appears that PN synchrony—in either spikes or pauses- may play a role in both time and rate coding in the CN neurons. However, it remains unclear what characteristic of “synchrony” in elaborate PN firing patterns can be represented in the downstream CN. Gauck and Jaeger (2000)[18] previously investigated the effect of PN synchrony on the rate modulation and spike timing of CN neurons using an experimental dynamic clamp approach. They found that PN synchrony plays an important role in controlling spike timing in CN neurons, but their study assumed that PN spike trains can be described as a Poisson process, which was later shown not to be true [12].

Based on the experimental study of pause synchronization [7], we defined in this computational work “synchrony” as synchrony of pauses with beginning or ending spikes synchronized, or, as overlapping pauses without spike synchronization. Using experimentally derived parameters for connectivity between the PNs and CN neurons and synthetic PN spike trains, we then investigated how these different types of simple spike synchrony influenced the firing response of the CN neurons. Further, we characterize the effect of PN firing rate preceding the synchronous pause on CN neuron’s firing both during and after the synchronous pause.

## Results

### CN neuron’s response to synaptic input

The firing of deep CN neurons is highly regular *in vitro* [18]. *In vivo*, the firing of these neurons becomes irregular [19] mediated through excitatory and inhibitory synaptic inputs. To match these *in vivo* data, we tuned both the gain of inhibitory and excitatory synapses impinging on the CN neurons so that the neurons fire spontaneously around 37–38 Hz, which is the observed mean firing rate *in vivo* of normal rats [19]. The analysis was performed for three model neurons corresponding to three different values of input gain: low, medium, and high, respectively. The gains of excitatory and inhibitory conductance are a specific percentage of their maximal synaptic conductance. The value of inhibitory gain for low, medium and high gain levels was 10%, 70% and 150%, respectively. For each gain level, the excitatory gain value was adjusted so that the model fires at levels of the aforementioned in vivo firing rate [19]. The excitatory gain values corresponding to low, medium and high gain levels were 7%, 12% and 24% respectively. Note that for each value of input gain, the analysis was performed in turn for each of the three models differing in rebound conductance profile (see Methods).

**Fig 1:**
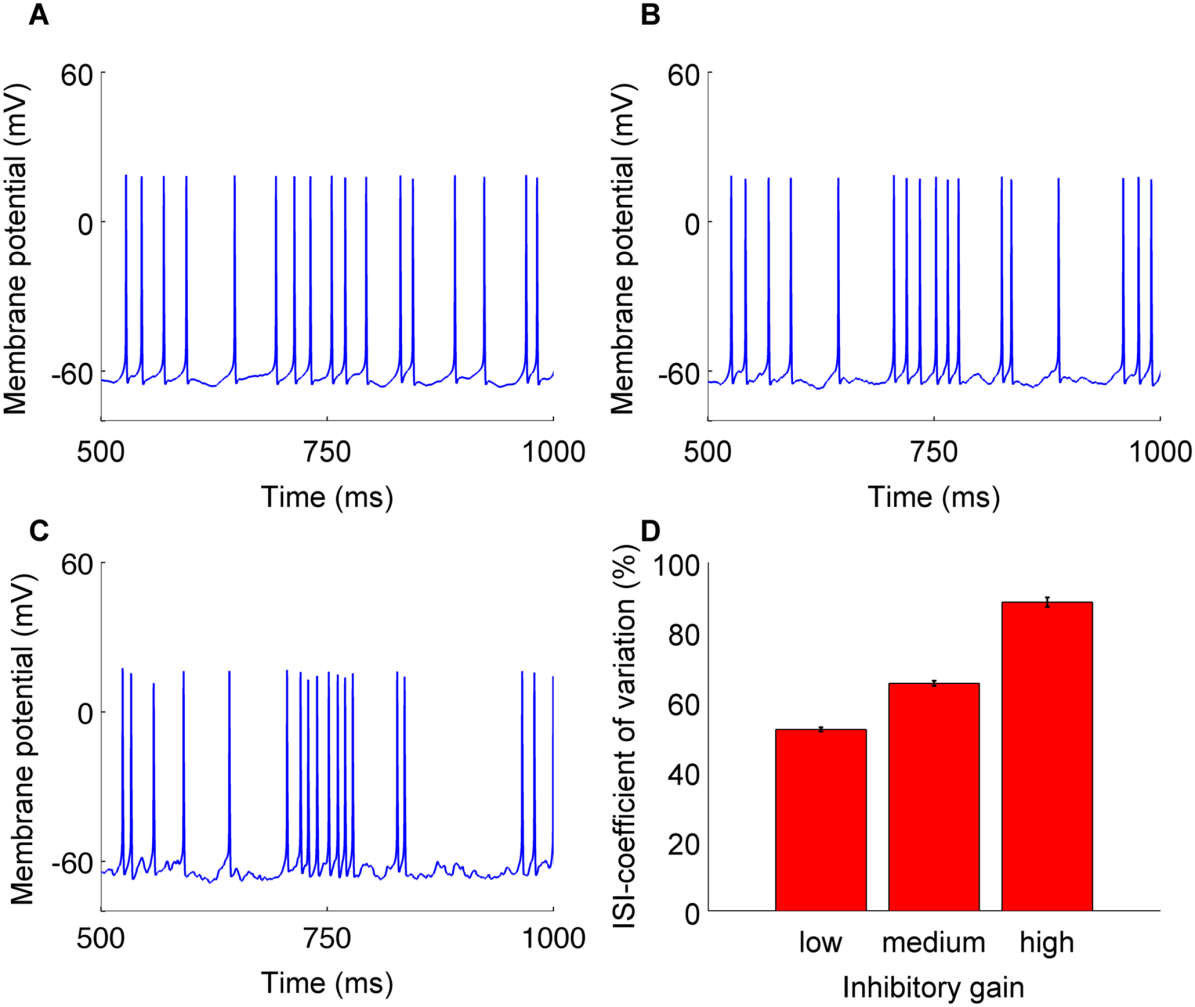
CN neuron’s firing pattern in response to synaptic inputs. Firing pattern of the CN neuron in response to same synaptic input sequence with low (A), medium (B) and high values of input gain (C), respectively. D: Coefficient of variation in inter-spike intervals of CN neuron’s spiking in response to synaptic input of varying gains.

Synaptic input had maximum impact on the regularity firing of the neuron at the high gain condition (Fig 1A–1C). A CN neuron’s spike train became more irregular as the gain of the synaptic input increases, as indicated by the increasing coefficient of variation in the inter-spike interval (Fig 1D). As CN neurons exhibit irregular firing pattern *in vivo* [19], the high gain condition observed in our study best matches CN firing in awake animals.

**Fig 2:**
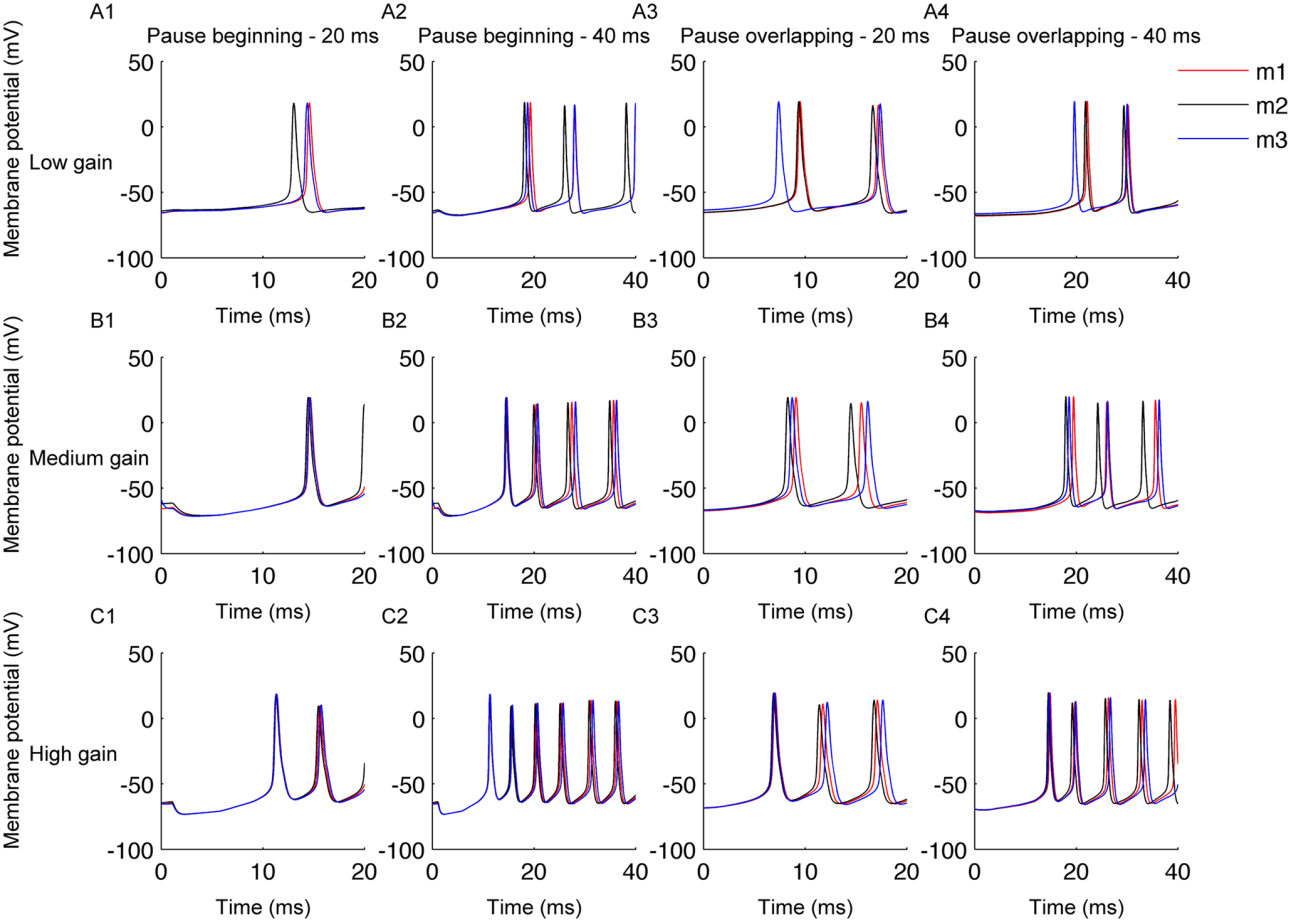
Membrane potential traces of three types of CN neuron (m1, m2, m3) during synchronous PN pauses for various input regimes. A1–A4: Membrane potential traces of three types of CN neuron for low gain condition. A1-Pause beginning synchronization 20 ms, A2-Pause beginning synchronization 40 ms, A3-Pause overlapping synchronization 20 ms, A4-Pause overlapping synchronization 40 ms. B1–B4: Same for medium gain condition. C1–C4: Same for high gain condition. Each rebound model is shown by a different color trace as indicated by the legend.

**Fig 3:**
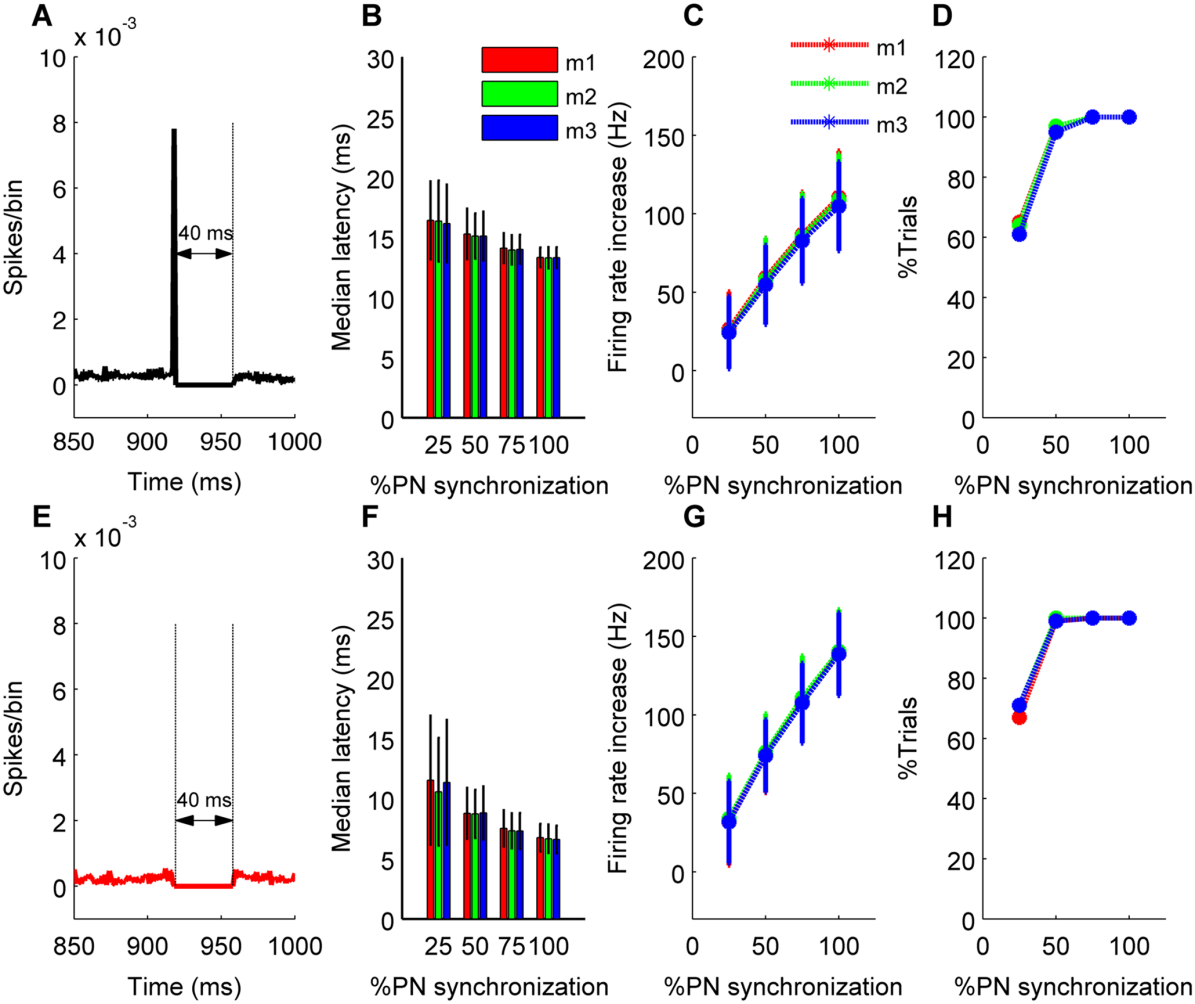
CN neuron’s response to synchronous PN pauses with pause length of 40 ms for pause beginning type synchronization (C-D) and pause overlapping type synchronization (F-H) (high input gain condition). (A&E) Population spike timing histogram of all PNs projecting onto the CN neuron. (B&F) Median latency of CN neuron’s spiking in response to the pause beginning type synchronization (B) or pause overlapping type synchronization (F) condition for 100 trials and three different models m1, m2, m3. (C&G) Increase in firing modulation of CN neurons during the synchronous pause calculated from 100 trials and three different models m1, m2, m3 for the two synchrony types. (D&H) Percentage of trials showing increase in firing rate quantified as reliability of increase for the two synchrony types.

In order to analyze the influence of PN synchrony on the firing response of the CN neuron, we characterized the neuron’s response to synchronous PN pauses (see Methods). Simulations were run with different synchrony types (pause beginning, pause ending or pause overlapping), with varying amount of input synchronization and different pause length and input gain. This analysis was carried out in all three models differing in rebound conductances (m1, m2, m3). The CN neuron’s response to synchronous pauses of length 20 ms and 40 ms for various values of input gain can be seen in Fig 2. Fig 3B–3D shows the response of the model neuron to 100 different trials where we quantified the accuracy of time-locking (Fig 3B), extent of increase in rate modulation (Fig 3C), and reliability of increase in firing (Fig 3D) for pause beginning synchronization. The figure represents the condition for high gain. Fig 3E–3H illustrates the results of pause overlapping synchronization. Increase in input synchronization resulted in increased rate modulation and an improved reliability of this increase, which will be further analyzed in the subsequent sections. Since most of the results were identical for all the three different rebound models (Fig 3), the subsequent figures in this study will reflect observations from only one of those models (m2).

### Effect of PN pause synchrony on the rate modulation of CN neurons

**Fig 4:**
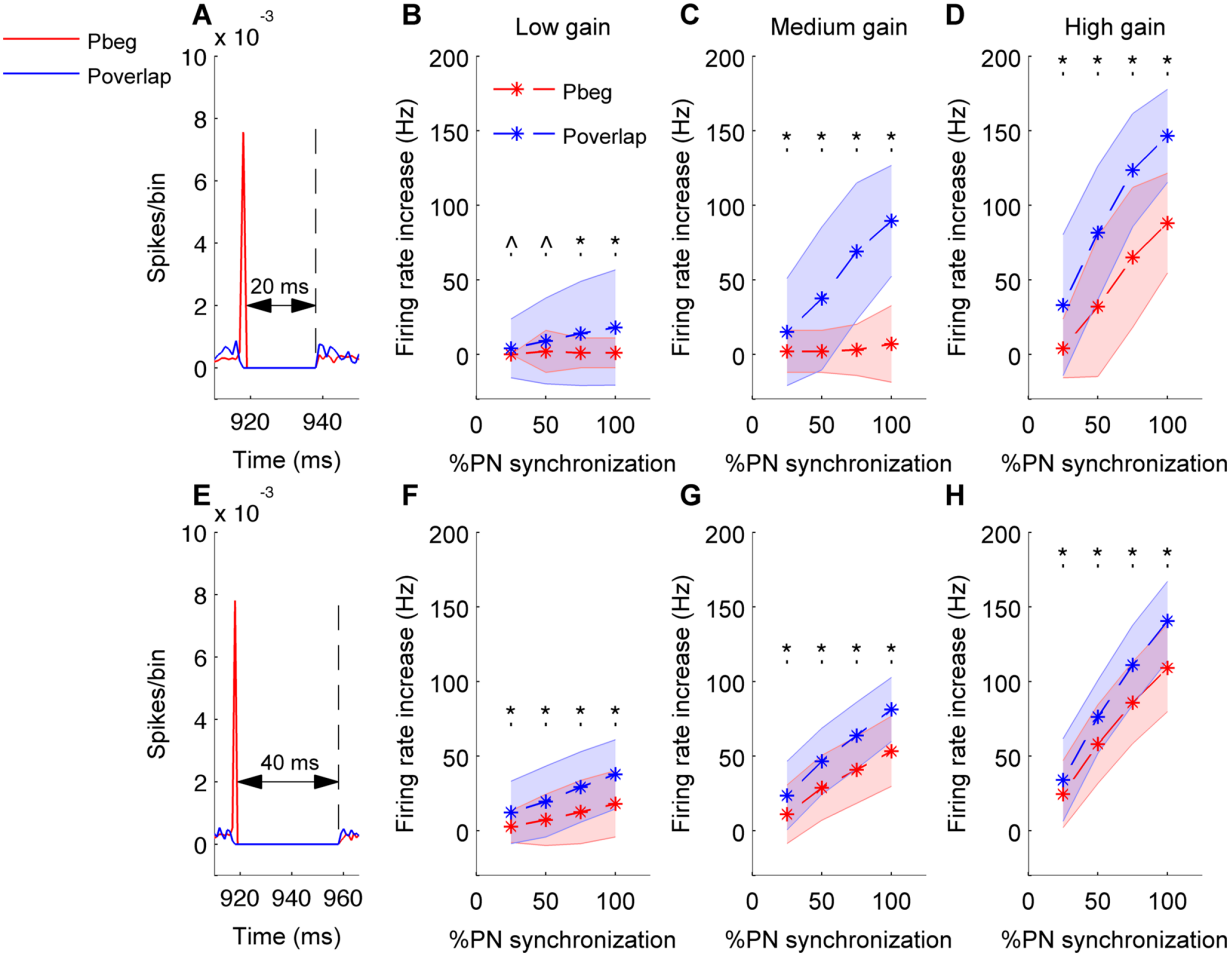
Pause overlapping synchronization mediates greater firing rate increases than pause beginning synchronization. Analysis for 20 ms (A-D) and 40 ms (E-H) synchronous pauses. In all panels red color stands for pause beginning condition, blue for pause overlapping condition. Error bars are represented by shaded region. (*) represents comparisons between pause beginning and pause overlapping type synchronization that are significant (p<0.05) and (^) represents insignificant comparisons (p> = 0.05). (A&E) Population spike timing histogram of all PNs projecting onto the CN neuron. One can notice synchronous pause of 20 ms (A) or 40 ms (E) for both types of synchronization. (B&F) Mean and standard deviation of increase in firing rate of CN neuron quantified for both pause beginning and pause overlapping conditions and for low input gain. (C&G) Same for medium input gain. (D&H) Same for high input gain.

We quantified the increase in firing rate of the CN neuron during synchronous pauses with spikes synchronized at the beginning or ending, or overlapping pauses (where no spikes are synchronized), for different amount of synchronization. Synchronous pauses were generated by randomly selecting a pause greater than threshold and temporally aligning it with pauses from other Purkinje neuron spike trains according to the desired amount of synchronization. Pause ending and pause overlapping synchronization consistently mediated greater firing rate increases compared to pause beginning synchronization. This could be seen for both pause values of 20 and 40 ms (Fig 4 and S1 Fig). For all synchronization types, the rate modulation increased with an increase in input gain for all values of synchrony. For example, increase in firing rate produced by 50% and 100% input synchronization increased by approximately 50 Hz and 91Hz respectively, from the low input gain (Fig 4F) to the high input gain (Fig 4H) condition (pause value of 40ms, pause beginning synchronization). From Fig 4B one can observe that, for both synchrony types a 20 ms pause is too small to mediate any firing rate increase for 25% and 50% of PN synchronization (low input gain condition). Hence for the same conditions, the modulation mediated by these two synchrony types is very small and doesn’t significantly differ from each other (p>0.05). For a pause length of 40 ms, the rate increases exerted in the pause overlapping or pause ending condition is significantly greater than that of pause beginning for all values of synchronization and all values of input gain (p<0.0041, Fig 4F–4H and S1 Fig). The increase in firing rate of the CN neuron exerted by pause ending type synchronization was not significantly different to that of pause overlapping condition for all values of pause length, input gain, and synchronization (p>0.15) (S1 Fig).

We also simulated the effect of a mixed-type synchronization where PN pauses were synchronized with all three types of synchronization (beginning, end and overlapping) in equal proportion. This resulted in increased firing rate modulation of CN neurons, which was greater than that of the pause beginning synchronization, but less than the pause overlapping condition.

**Fig 5:**
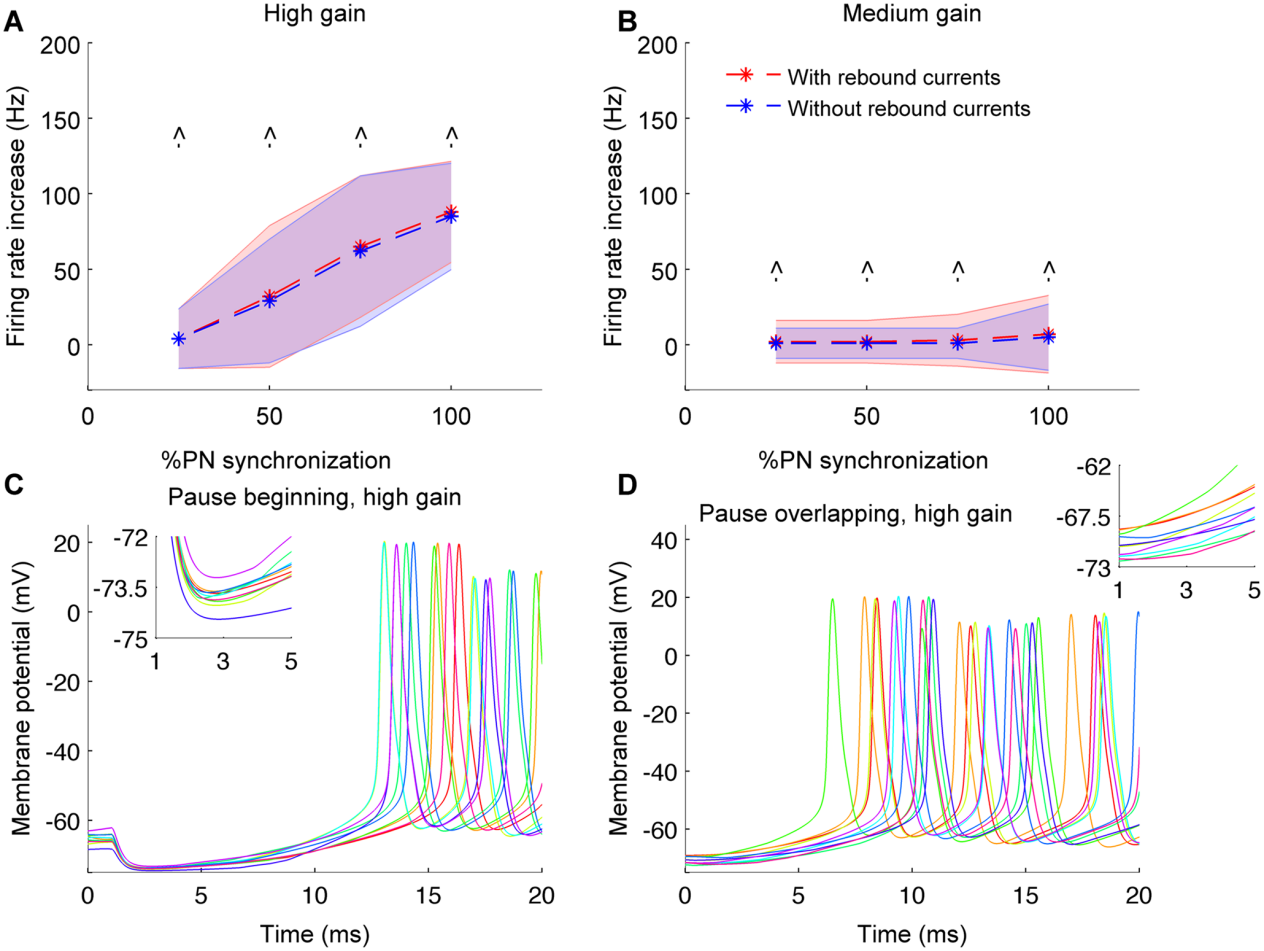
Mechanisms underlying increased CN neuron's firing rate during synchronous PN pauses. (A) Increase in firing rate of CN neurons quantified with and without the rebound conductances in the model (Pause beginning, high gain condition, and synchronous pause length of 20 ms). (B) Same as in panel A but for medium gain condition. (C) CN neuron's membrane potential traces during synchronous pause of length 20 ms and pause beginning synchronization with 100% PN pauses synchronized (high gain condition). Traces from 10 trials plotted. Inset shows the deep hyperpolarization mediated by pause beginning spikes during the beginning of synchronous pause (1 to 5 ms). (D) Same as in panel C but for pause overlapping synchronization.

In order to elucidate the membrane mechanisms underlying increased firing of CN neurons during the synchronous PN pauses, we blocked the rebound conductances (namely HCN channel, T-type calcium channel and persistent sodium channel) in the CN neuron model. Fig 5A and 5B shows that when the rebound conductances were blocked, the CN neuron's firing rates within the synchronous PN pause were not altered (pause beginning synchronization, pause length = 20ms). Therefore, the increase in firing rate during synchronous PN pauses is mediated by a passive increase in membrane potential until the CN neuron's firing threshold is reached, at which point fast sodium currents are activated, resulting in spiking activity. The lower increase in firing rates caused by pause beginning spike synchrony is explained by the deep hyperpolarization of the CN neuron’s membrane up to the chloride reversal potential (at -75 mV) (Fig 5C), causing the rebound to take longer as compared to the pause ending and pause overlapping conditions. As a result, the spike threshold will be reached later, and the average firing rate measured over the pause duration is therefore lower than that of the other pause synchronization conditions. For all types of synchrony the increase in firing rate was restricted to within the period of synchronous pause and no substantial increase was observed outside the pause period.

Next we investigated the effect of the amount of synchrony on the rate modulation of CN neurons. Increase in PN input synchronization resulted in an increased rate modulation for all pause synchrony conditions (S2 Fig). We start by describing the observations related to pause beginning synchronization. Firing rate increase mediated by pause beginning type synchronization increased from 24.5 Hz to 109 Hz for 25% to 100% input synchronization respectively (S2–S2D Fig 40 ms pause). For the same synchrony condition and pause length, the amount of synchrony is represented as significant rate increases for medium and high values of input gain (S2C and S2D Fig p<0.0001) but not for low input gain (S2B Fig. p>0.1). Input synchrony for the 20 ms pause length is represented as significant rate increases for the high input gain condition (S2D Fig) (p<0.0045) but not for low and medium gain conditions (S2B and S2C Fig p>0.1). Therefore, the amount of input synchrony is better represented in the firing rate increases of CN neurons by a 40 ms synchronous pause than by a 20 ms synchronous pause.

We observed similar results for the pause overlapping synchronization condition: a 40 ms pause represents the amount of presynaptic PN pause synchrony better (S2E–S2H Fig). One notable difference is that input synchrony of the 40 ms pause sequence is significant even for low input gain condition (S2F Fig p<0.005). Thus, we conclude that for both synchrony types the amount of synchrony is better represented in downstream CN neurons when the PN to CN neuron synapses have a high gain because the release from stronger inhibition produces a bigger increase in firing modulation.

### Time-locking of CN neuron spikes to synchronous PN pauses

According to Person and Raman, PN synchronization causes brief periods of relief from inhibition and this time-locks the CN neuron’s response to the input synchronization [5]. This novel form of time coding, which transmits the timing of occurrences of PN synchronization to its downstream targets was suggested to be present both in *vivo* and in *vitro* [5]. By varying the amount of pre-synaptic PN synchronization, different pause length (20 or 40 ms), and synchrony type (pause beginning or pause overlapping), we investigated the effect of pause synchrony on time-locking of the CN neuron spikes.

**Fig 6:**
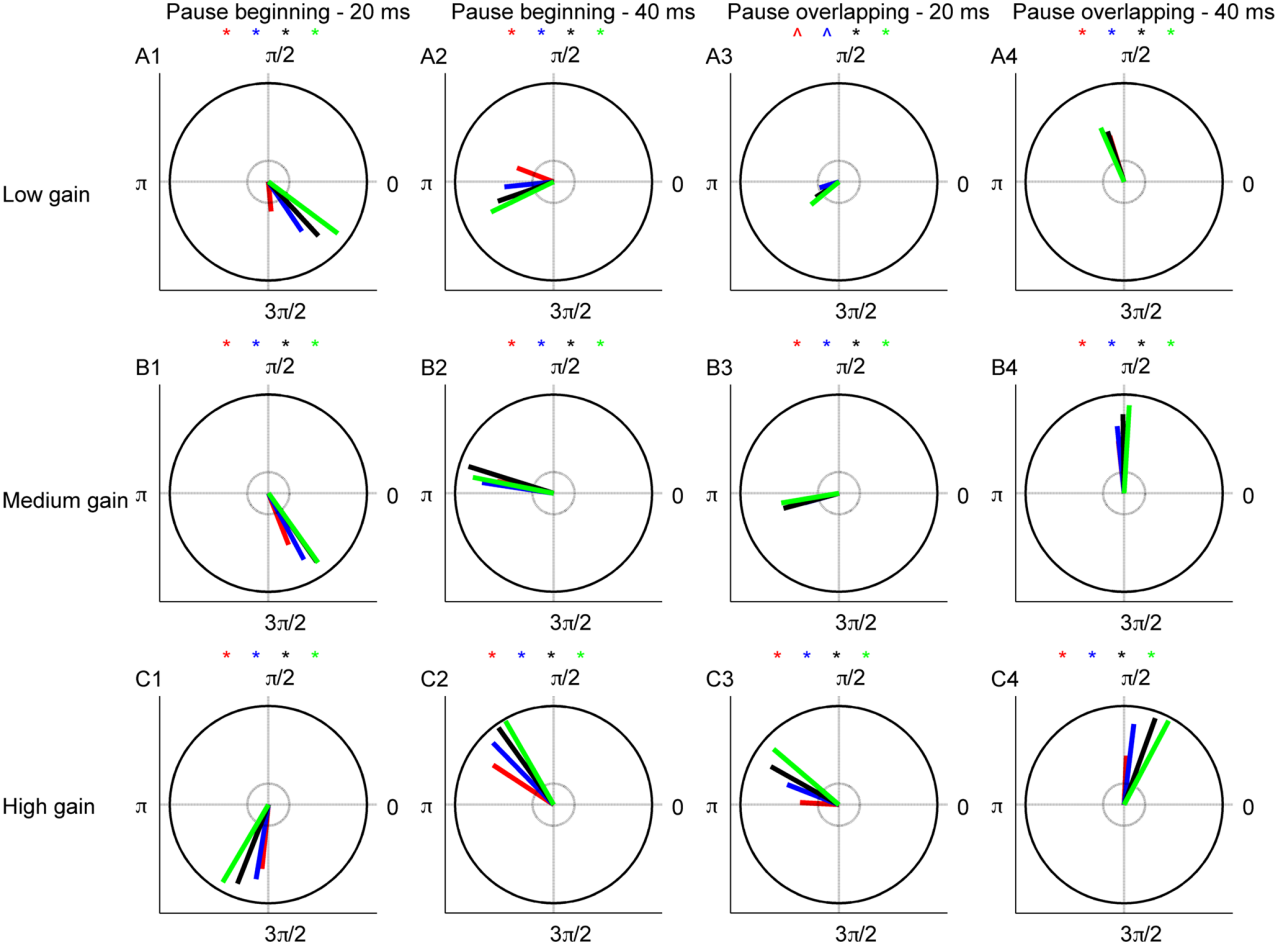
Time-locking of CN neuron spikes quantified using circular statistics. The entire circle is the duration of the synchronous pause. The vector strength computed by vector addition from 100 different trials is shown by red, blue, black and green lines for 25%, 50%, 75% and 100% synchronization. respectively. (*) is significance marker determined by the Rayleigh statistic. The small grey circle indicates the threshold of vector strength determined by Rayleigh statistic. (A1–A4). Circular plot of vector strength in the synchronous pause for low input gain condition (A1-pause beginning 20 ms, A2-pause beginning 40 ms, A3-pause overlapping 20 ms, A4-pause overlapping 40 ms) (B1–B4). Same for medium input gain condition. (C1–C4). Same for high input gain condition.

Synchronous pause beginning spikes effectively time-locked the CN neuron’s response to the synchronized event (Fig 6). For almost all values of input gain and amount of synchronization, the value of vector strength obtained was significant (p<0.01, Fig 6). Increased input pause synchronization decreased the variability in latency, increased the vector strength and hence the precision of the time-locking phenomenon. For example, for a pause length of 20 ms and pause beginning synchronization, the vector strength for 25%, 50%, 75% and 100% synchronization are 0.65, 0.76, 0.85, 0.91 (p<0.01) respectively (Fig 6C1, high gain condition). Brief releases from inhibition produced by the synchronous pause and the deep hyperpolarization of the neuron by the pause beginning spikes caused effective time-locking of CN neuron spikes to the input event. This effect was enhanced at the high input gain condition. Pause overlapping synchronization also time-locked CN neuron’s spiking with better time-locking observed for increased input synchronization. As for pause beginning synchronization, the vector strength for pause overlapping type synchronization was significantly greater than from uniform distribution of spike latencies on a unit circle except for some low gain conditions (p<0.01 Fig 6). Thus brief synchrony of PN pauses time-locks the CN neuron’s spiking. The latency distributions for pause ending synchronization were similar to those for pause overlapping type for all values of gain and pause length (p>0.12) (S3 Fig).

### Synchronous pause beginning spikes mediate more accurate time-locking of CN neuron’s spiking to inhibition

**Fig 7:**
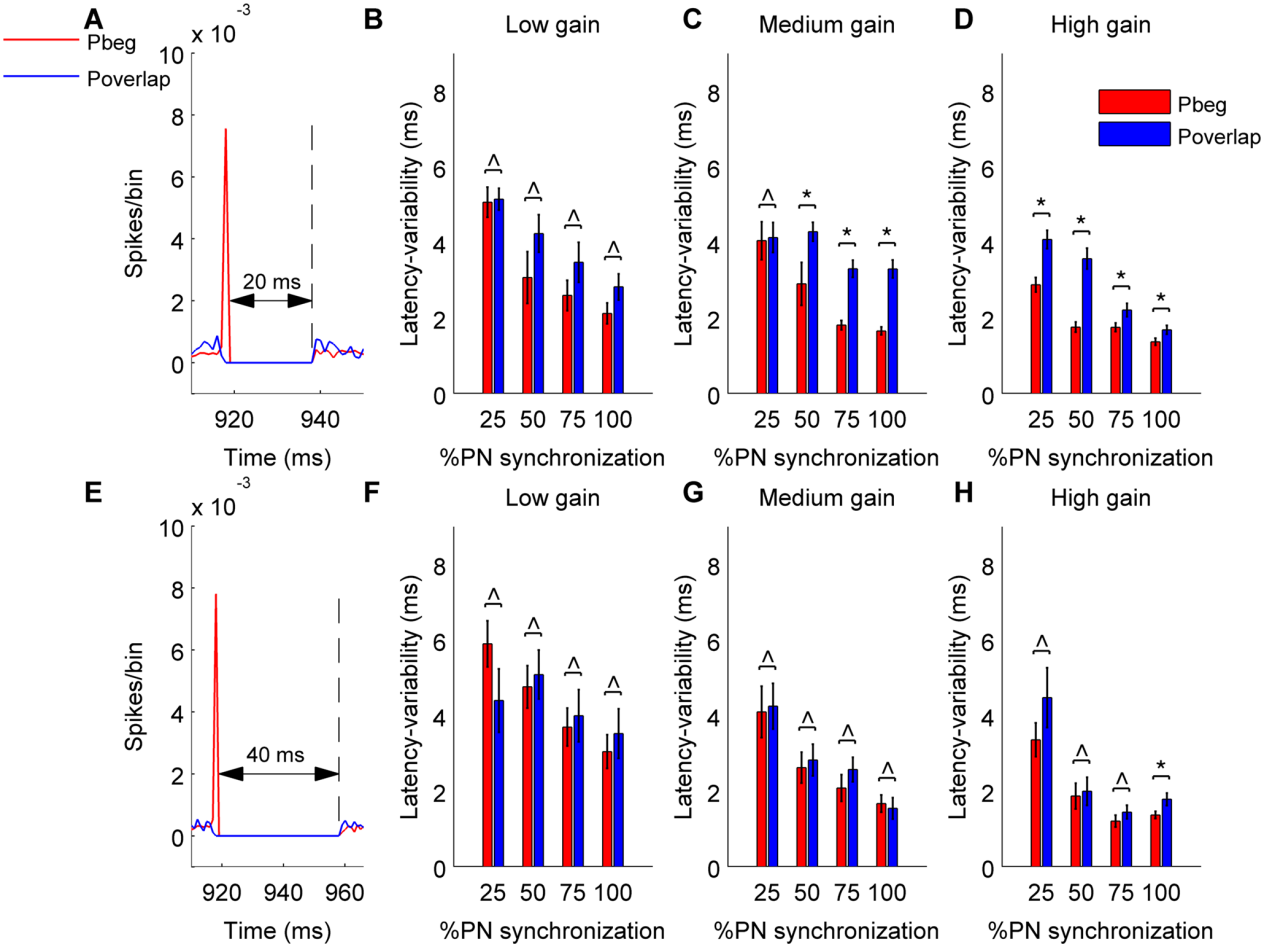
Pause beginning type synchronization mediate more accurate time-locking than pause overlapping synchronization. In all panels red color stands for pause beginning condition, blue for pause overlapping condition. (*) represents comparisons in variability of latency between pause beginning and pause overlapping type synchronization that are significant (p<0.05) and (^) represents insignificant comparisons (p> = 0.05). (A&E) Population spike-timing histogram of all PNs projecting onto the CN neuron. (B&F) Variability of latency calculated from 100 trials for pause beginning and pause overlapping type synchronization and low gain condition. (C&G) Same for medium gain condition. (D&H) Same for high gain condition.

In the previous section we demonstrated how synchronous PN pauses elicit time-locked CN neuron spikes thereby transmitting the timing information in a reliable manner to downstream targets of CN neuron. We compared the degree of time-locking caused by pause beginning or pause overlapping synchronization by looking at their spike latency distributions in detail. This analysis revealed that time-locking by pause beginning spikes is more accurate than that of pause overlapping condition for a 20 ms (Fig 7A–7D) synchronous pause but not for a 40 ms (Fig 7E and 7F) pause period. However, this significant increase in the accuracy of time-locking between the two synchronization types for a 20 ms pause sequence is seen only for high gain condition (Fig 7D, p<0.01, synchronous pause length = 20 ms). For low gain the increase in the precision of time-locking between the two types of synchronization is not significant (Fig 7B, p>0.09) and for medium gain the increase is significantly seen for 50%, 75% and 100% input synchronization (Fig 7C, p<0.01) but not for other values of input synchronization (p = 0.42). With a 40 ms pause the effect of the asynchronous PN inhibition becomes more dominant, resulting in a significant effect for 100% synchronization only. In conclusion, pause beginning type synchronization can cause better time-locking than pause overlapping synchronization depending on the strength and synchrony of inhibition.

**Fig 8:**
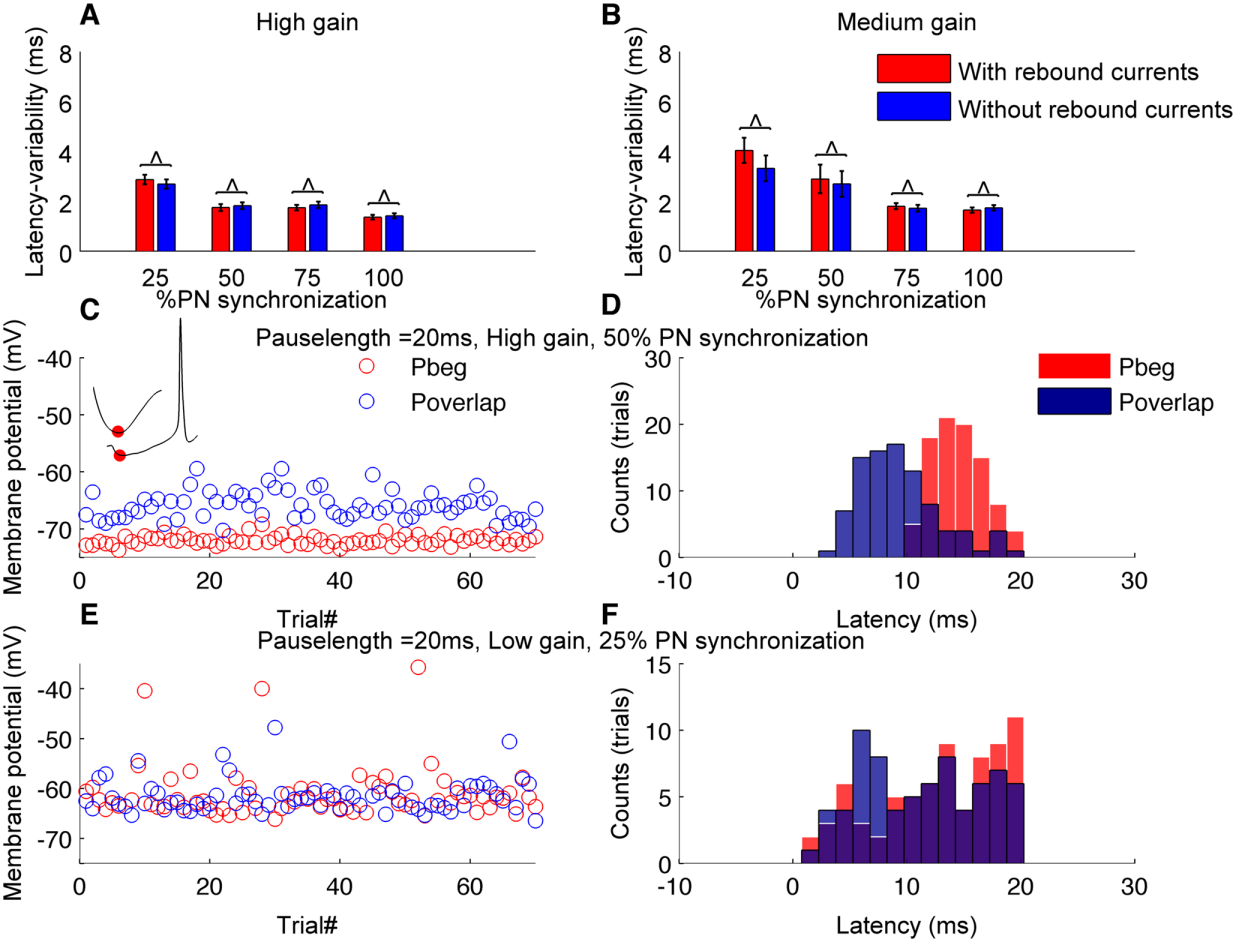
Mechanisms underlying time-locking of CN neuron spikes to synchronous PN pauses. (A) Variability of latency calculated from 100 trials with and without the presence of rebound conductances (Pause beginning type synchronization, high gain condition and pause length of 20 ms). (B) Same as in panel A but for medium gain condition. (C) Scatter plot of CN neuron's membrane potential during the deep hyperpolarization mediated by synchronous pause beginning spikes (red) or the pause overlapping condition (blue). High gain, 50% synchronization, pause length of 20 ms. Inset represents the response of CN neuron during pause beginning synchronization during a single trial. The deep hyperpolarization during initial part of the synchronous pause is given in the zoomed view of the inset. Red dot represents the recorded membrane potential for that trial. (D) Comparison of distribution in latency between pause beginning and pause overlapping synchronization condition (n = 100). (E, F) Same as in C, D but for low gain, 25% synchronization and pause length of 20 ms (n = 130).

In order to elucidate membrane mechanisms causing the time-locking of CN neuron spiking to PN pause synchronization, we blocked the rebound conductances in CN neuron model and analyzed its effect on time-locking. Rebound conductances had an insignificant effect on the time-locking of the CN neuron (Fig 8A and 8B). There was no significant change in the variability of the latency when membrane rebound conductances were blocked (p>0.31). Instead, we attribute the time-locking to release from inhibition (Figs 5C and 5D, 8C and 8E), as has been previously demonstrated in other systems [20]. In the presence of strong inhibition, synchronous pause beginning spikes hyperpolarize the neuron's membrane potential and push it to a common state near the chloride reversal potential (-75 mV) for every trial (Fig 8C). As a result, the time it takes to ramp up from that potential is always the same and hence precise time-locking is achieved and observed as a narrow distribution of spike latencies, Fig 8D. In pause overlapping and pause ending conditions, the release of inhibition is not orchestrated as such and hence the membrane potential is different across trials, which results in differential times to ramp up to firing threshold. As a consequence, the latency of the first spike is noisier and precise time-locking is reduced (Fig 8D). With low inhibition, even pause beginning synchrony fails to hyperpolarize the membrane potential (Fig 8E) to the chloride reversal and spike latencies become noisier, which reduce precise time-locking (Fig 8F).

### Strong inhibition increases the jitter tolerance level of CN neurons

Up to this point, we used precise synchrony of spikes without any jitter. Because this is a rather idealized situation, we also investigated whether jittered pause spikes can still evoke time-locking of CN neuron’s responses. Therefore, we quantified the time-locking behavior of the neuron when presented with synchronous pause differing in the amount of synchronization and pause beginning spikes jittered with various values of jitter. We generated synchronous PN pauses in the same way as described in previous sections but with jittered pause beginning spikes and a pause duration of 40 ms.

**Fig 9:**
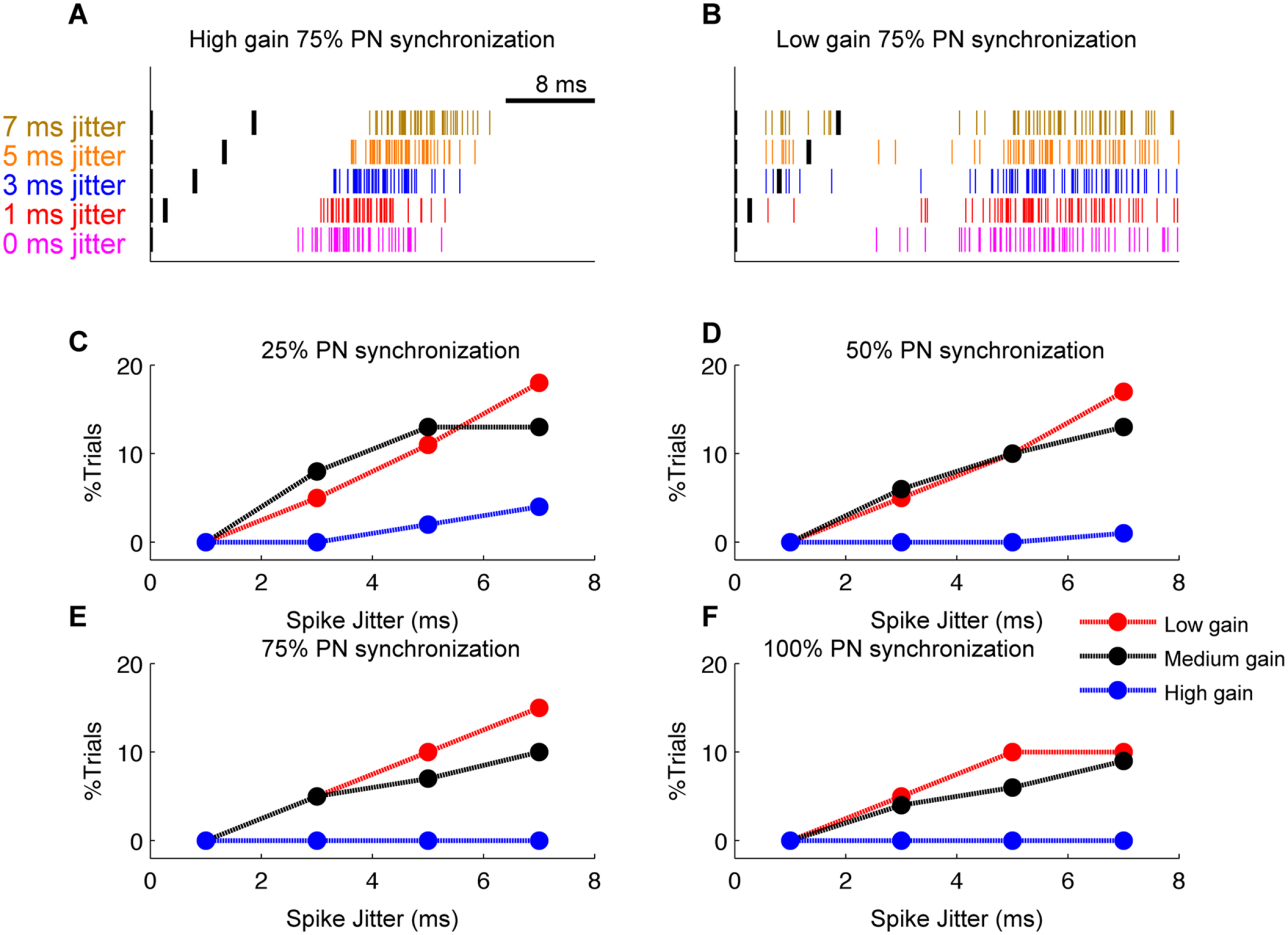
Stronger inhibition enables the CN neuron to transmit the timing information accurately even in the presence of significant PN spike jitter. Analysis is for pause beginning type synchronization and a pause value of 40 ms. (A) Raster plot of CN neuron’s first spike during the synchronous pause from 100 trials for high gain condition (75% PN synchronization for various values of spike jitter). Black lines indicate the spike jitter period. (B) Same as in panel A but for low gain condition (75% PN synchronization). (C) Percentage of trials where CN neuron's first spike during the synchronous pause is within the jitter period when 25% PN pauses are synchronized. (D) Same when 50% PN pauses are synchronized. (E) Same when 75% PN pauses are synchronized. (F) Same when 100% PN pauses are synchronized.

When the CN neuron's input gain is high, the strong inhibition prevents the neuron to spike during the jitter period. This can be seen in Fig 9A where, for high input gain and 75% PN synchronization, the neuron's first spike post synchronized pause onset is only after the complete jitter period. Also, for the same input condition, CN neuron's first spike latency increased with jitter period, but actually decreased when measured relative to the end of the jittered spikes. For example, the median latency decreased from 13.38±1.23 ms for 1 ms spike jitter to 10.98±1.16 ms for 7 ms spike jitter.

For the low gain condition, the CN neuron often fired during the jitter period because the inhibition was too weak (Fig 9B). We compared the spike timing accuracy of CN neuron for various values of spike jitter by analyzing the distribution of first spikes within the synchronous pause (from all trials) post the jitter period and within the jitter period separately. The variability of spike latency post jitter period was not significantly different from each other when compared between different spike jitter conditions. We observed this for all values of input gain and input synchronization. In contrast, the percentage of trials where the neuron spikes during the jittered period increased as the amount of spike jitter increased for low gain condition. For 75% PN synchronization this value increased from 5% to 15% from a spike jitter value of 3 ms to 7 ms (Fig 9E). Therefore when the strength of PN inhibition is weak, spike-timing precision decreases as the amount of the spike jitter increases. But when PN inhibition is strong, the spike timing precision of CN neuron is the same for all values of spike jitter as the neuron allows its first spike only after the jitter period. Thus, stronger inhibition enables the CN neuron to transmit the timing information accurately even if there is a significant jitter in the incoming spikes.

### CN neuron’s rebound responses are modulated by PN firing

So far our analysis was limited to the effect of synchronous pauses on the firing modulation or time-locking within the pause. Our analysis has revealed that, on average, rebound conductances have no or little effect on this firing modulation or time-locking. CN neurons exhibit rebound firing when the membrane is hyperpolarized for a considerable amount of time and subsequently released from it [21]. De Schutter and Steuber [17] suggested that the amplitude of the CN neuron rebound bursting (in this study measured as the CN neuron’s firing rate for a duration of 1s from synchronous pause onset) is directly proportional to the PN firing rates before the synchronous pause. This can be explained by the inactivation/de-inactivation characteristics of rebound conductances (persistent sodium current, T-type calcium current). The rebound conductances that are inactivated during spiking activity of the neuron require substantial membrane hyperpolarization to get de-inactivated [21]. Therefore, high PN firing preceding the synchronous pause leading to more hyperpolarization of the CN neuron's membrane may cause more de-inactivation of rebound conductances and hence higher amplitude of rebound responses of CN neuron. We employed cross-correlation analysis to study the effect on PN firing preceding the synchronous pause on the amplitude of CN neuron's rebound responses.

**Fig 10:**
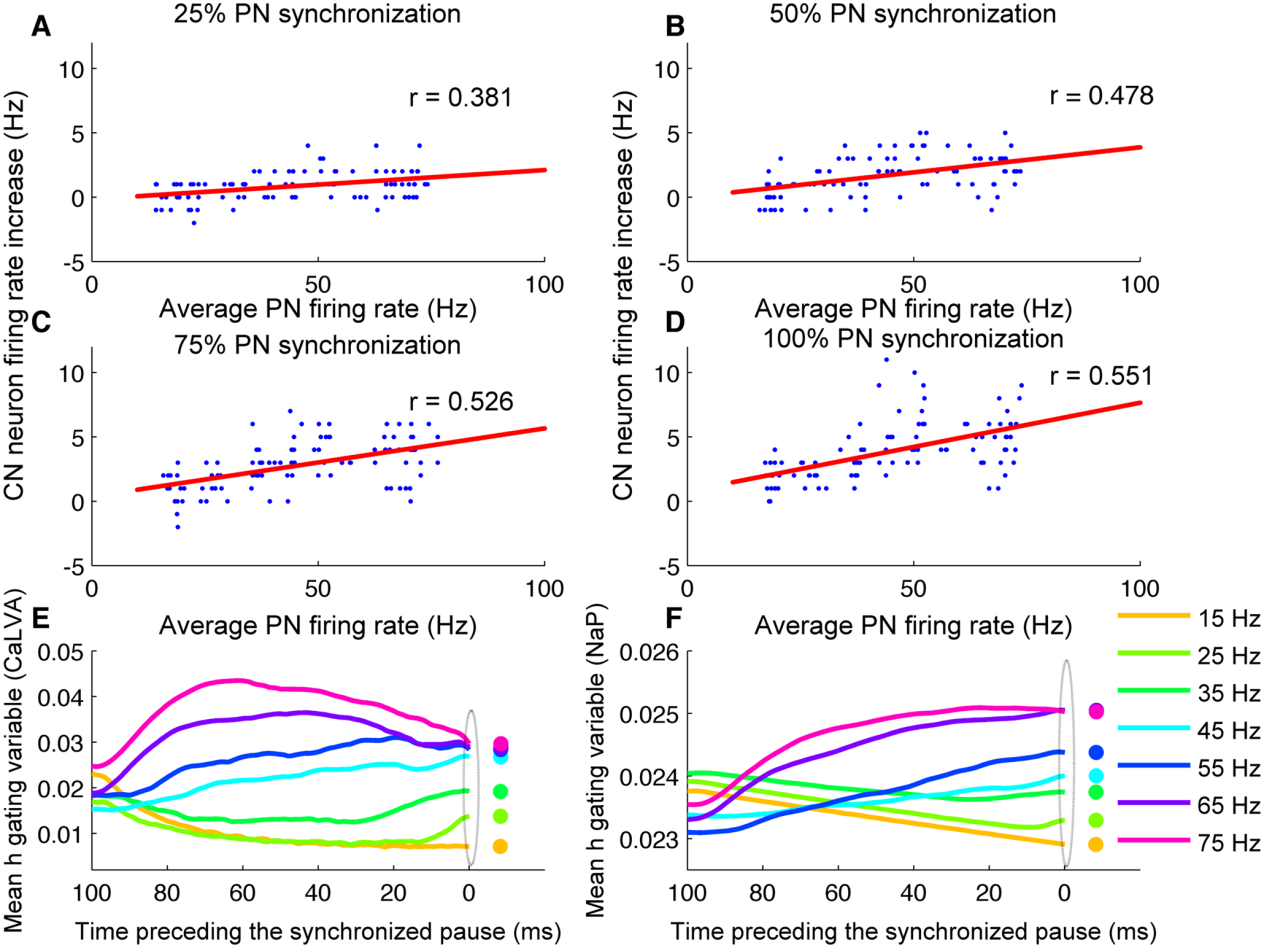
PN firing rate preceding the pause controls the amplitude of CN rebound firing. Panels A-D: CN neuron’s firing rate increase post synchronous pause onset plotted as a function of average PN firing rate preceding the pause for 25%, 50%, 75% and 100% of input synchronization respectively for high gain condition. Panel E: Mean inactivation gating variable of T-type calcium current (CaLVA) for 100 ms preceding the synchronous pause (calculated from 14 trials of each value of PN firing rate preceding the pause, 100% PN synchronization). Panel F: Same as panel E for inactivation of persistent sodium (NaP) current.

Average PN firing rate (from all PNs participating in the pause synchrony) during a period of Δt before the synchronous pause was forced to a particular frequency 'fPN' (see METHODS). We sampled a uniform distribution of fPN from various trials to avoid sampling biases associated with particular PN firing frequencies. CN neuron’s firing rate was quantified for a period of 1s starting from synchronous pause onset. We observed only small increases in the frequency of CN neuron firing in response to increasing average PN firing rate (Fig 10A–10D). Maximum firing rate increase of CN neurons post pause onset increased from 4 Hz to 11 Hz for 25% to 100% input synchronization (Fig 10A–10D). Our results are consistent with that of experiments in mouse cerebellar slices where CN neurons respond to 150 ms, 100 Hz stimulation of presynaptic PN afferents with a moderate post inhibitory firing rate increase of 12 Hz [22]. The absence of larger firing rate increases is due to the limitation imposed by chloride reversal potential of GABAergic currents [22] and the requirement of substantial membrane hyperpolarization to de-inactivate the rebound currents [21,22]. The steady state half inactivation value for T-type calcium current and persistent sodium current is around -80 mV [21]. But PN neuron inhibition of CN neurons is limited by vivo chloride reversal potential whose value in CN neuron is around -75 mV [22]. Therefore the rebound conductances exhibit only partial recovery from inactivation during PN inhibition. However, even with this level of recovery from inactivation, the de-inactivation of rebound conductances were directly modulated by the PN firing rate preceding the synchronous pause (Fig 10E and 10F). The greater the firing rate of PNs before the synchronous pause, the greater the recovery from inactivation. Cross-correlation of PN and CN neuron firing rate increase revealed a high significant value for all values of input synchronization. The pearson correlation r increased from r = 0.381 in the 25% synchronicity case to r = 0.551 in the 100% synchronicity case. Thus the firing rate of PNs before the pause controls the amplitude of succeeding CN rebound firing.

## Discussion

In this study we investigated the effect of PN synchrony in the context of pauses on the spiking response of CN neurons. We stress the importance of pause synchrony in our simulations because the cerebellum is known to control the motor output pattern by transient disinhibition of CN neurons through corresponding pausing of PN firing [23,24]. A wide range of motor behavior can be elicited by transient suppression and disinhibition of PN spiking and CN neuron output, respectively [25,26]. Further, CN neuron’s spikes are elicited by transient decrease in PN inhibition of minimum 15 ms duration [18], see also [25].

We find that pause overlapping and pause ending spike synchronies mediate greater firing rate modulation during the synchronous pause than their pause beginning counterparts. It is worth noting that the synchrony types are not uniquely encoded in the firing rate of these neurons as their standard deviations overlap (Fig 4). Similar to an *in vitro* study [18], we also find that the amount of input synchrony modulates the firing of CN neurons with increased input synchrony resulting in an increase in the firing rate of CN neurons. How could the cerebellar system effectively make use of the increase in a CN neuron’s firing rate mediated by the amount and type of PN pause synchronization? Recently, an *in vivo* optogenetic study highlighted the importance of input synchrony through transient ‘pausing’ of PNs by photostimulation of molecular layer interneurons expressing channelrhodopsin [25]. They show that various parameters of eye-blink movement kinematics (amplitude, speed of movement) could be modulated by the amount of suppression of PN firing (number of PNs participating in the pause) and corresponding modulation of firing rates in CN neurons. Therefore, the effect of amount of input synchrony observed in our simulations is directly related to control of movements and behavior in awake, behaving animals.

CN neurons exhibit both rate and time coding, but it remains unclear how these neurons can switch between these two different coding schemes. We reasoned that synchronous pause length could play a vital role in controlling the coding strategy of CN neuron. Therefore we compared the effect of pause length, by measuring the effect of synchronous pauses of 20 or 40 ms. For pause beginning synchronization, a 40 ms pause sequence evoked greater firing rate increases than a similar 20 ms pause sequence (S4F–S4H Fig). Conversely, a 20 ms pause sequence was more accurate in transmitting the timing information compared to a 40 ms pause (S4B–S4D Fig). So, pause duration plays a vital role in promoting rate or time coding of the CN neuron with increasing pause length resulting in increased rate modulation and decreased accuracy of time-locking of the CN neuron’s response to a synchronous pause.

With respect to time-locking of CN neuron spikes to PN pause synchrony, we found that pause beginning synchronization cause more accurate time-locking than pause overlapping or pause ending type synchronies. A similar phenomenon was reported earlier by Person and Raman [5] where they quantified the extent of time-locking of CN neuron spikes to PN spike synchronization. The time-locking mechanism mentioned in [5] requires synchrony of PN ISIs, the “ISIs” could either be a regular ISI or a pauses whereas the time-locking mechanism quantified in our simulations only deals with synchrony of pauses. Accordingly, the time-locking in our simulations is most likely caused by molecular layer interneurons, which provide powerful inhibition to nearby PNs. Complex spikes can also trigger pauses in simple spike firing of PNs, and complex spike induced pauses can be synchronized across PNs as a single olivary axon targets multiple PNs of the same Aldolase C expression [27]. Nevertheless, PN simple spike pauses outnumber the number of complex spikes by a factor of 25 (assuming 1 Hz complex spike frequency, 50% PN ISIs contributed by pauses and 50 Hz PN spontaneous firing rate)[7]. From Fig 3d of [5] one can infer that for any stimulus interval less than 20 ms (PN firing rates greater than 50 Hz), the “brief relief” from inhibition required for time-locking is insufficient so the neuron’s excitability has to be increased (by applying a DC current) in order to obtain a response. As such, and in line with our findings, it appears that time-locking is an inherent phenomenon of “longer” length ISIs or so called pauses. Two vital factors affect the precision of the time-locking mechanism: chloride reversal potential and amount of PN pause synchronization. A strongly hyperpolarized chloride reversal potential leads to stronger inhibition and better alignment of the CN neuron repolarization. Similarly, increased PN synchronization also leads to stronger hyperpolarization.

The amount of PN synchrony reaching a CN neuron is unknown [1]. This is because PN to CN projection patterns are not completely understood [27,28]. Although it is known that CN neurons receive projection from PNs of the same zebrin signature (PNs in aldolase C positive compartments project to caudoventral CN and those in negative compartments project to rostrodorsal CN) [28], little is known about the spatial locations of PNs projecting to specific CN neurons. Anatomical tracing studies indicate that terminal arbors of PNs located in the same aldolase C compartments can be wide, and significantly separated in the downstream CN [28]. Moreover PN axons that are segregated mediolaterally within the same aldolase C compartment may project to non-overlapping areas in the CN [28]. These facts indicate the possibility of CN neurons receiving projections from PNs in multiple longitudinal compartments (of same aldolase C expression). Future research in this area may confirm whether CN neurons receive projections from PNs in single (same or different lobules) or multiple rostrocaudally oriented compartments (of the same aldolase C expression). Experiments measuring PN simple spike synchrony within and across various cerebellar regions (compartments, lobules) could reveal more information regarding spatial organization of PN synchronization.

We conclude that the multiplexed coding scheme [29] of the PN spike train proposed in [17] is preserved in the spiking response of CN neurons to synchronized PN pauses, i.e., the time locked latency being a timing signal and the CN neuron’s firing rate a rate code.

## Methods

### Cerebellar nucleus neuron model

The CN neuron model used in our study is based on a previously published model [21]. Three different instantiations of the model are used to mimic three different rebound spiking regimes (m1, m2, m3). Rebound responses in [21] were characterized by the presence of fast rebound burst and prolonged rebound spiking activity. m1 is characterized by the presence of fast rebound burst and a pause followed by prolonged rebound period (Fig 3, Neuron 1 of [21]). m2 is also characterized by the presence of fast rebound burst and a pause but followed by a very strong prolonged rebound period (Fig 3, Neuron 2 of [21]). Rebound response of m3 consists of fast rebound burst and a transition to prolonged rebound period without a notable pause (Fig 3, Neuron 3 of [21]). These rebound regimes were implemented in the model through specific combinations of HCN, T-type calcium channel and persistent sodium conductances. Because the results for these three models were nearly identical, we only show results obtained by model m2. The model rests at a temperature of 32°C. Through out the paper, HCN channel, T-type calcium channel (CaLVA) and persistent sodium channel are referred to as rebound conductances. All simulations were performed in NEURON version 7.3 [30].

### Inputs to the model CN neuron

CN neurons receive inhibitory inputs from PNs and excitatory inputs from mossy fibers. Both synaptic pathways are included in our model and described below. Climbing fiber inputs were not simulated.

### Inhibitory PN connections

PNs inhibit CN neurons. The number of PN synapses on a single CN neuron is highly debated and values in the literature range from 40 [5] to 860 [4]. We assumed a total of 200 PN connections on a single CN neuron [5,6,31–33]. These synapses were distributed uniformly on the CN neuron dendritic compartments. Synaptic conductance time course was modeled according to the following equation

$$G_{syn}\left(t\right)=\;gmax\times N\times \left[\text{exp}\left(\frac{-t}{\tau_{\text{decay}}}\right)-\text{exp}\left(\frac{-t}{\tau_{rise}}\right)\right]$$ (1)

where *τ*_*rise*_ and *τ*_decay_ are rise and decay time constant respectively. *gmax* is peak synaptic conductance, and N is a normalization factor that makes the maximum of *G*_*syn*_(*t*) equal to *gmax*.

PN to CN neuron IPSCs time constants are based on published experimental data [5]. IPSCs reported in this study were recorded at physiological temperatures and exhibit rapid decay with a decay constant of around 2.5 ms. We assumed a maximal synaptic conductance g_max_ = 11.7 nS and synaptic reversal potential of -75 mV [34]. The synaptic parameters of PN to CN neuron synapse used in the model are listed in supplementary information S1 Table). The rise and decay time constants of PN to CN neuron IPSCs were temperature corrected using experimentally determined Q_10_ values for synaptic current kinetics (Q_10_ = 2)[35,36]. The PN—CN neuron synapse is characterized by depression of IPSCs [34,37]. Synaptic depression reduces the synaptic conductance for subsequent pulses of a high frequency input train. Depression is not instantaneous but follows a time course reaching a steady state depression value. Synaptic depression was modeled by equations described in [12] and steady state depression data taken from multiple pulse depression values from experimental data (S1 Table). The equations for steady state release probability (*R*_*ss*_) and time constant of depression (*τ*) was fitted to experimental data [34]. The equations are given below:

$$R_{ss}\left(r\right)=\;0.08+0.60*exp\left(-2.84*r\right)+\;0.32*exp\left(-0.02*r\right)$$ (2)

τ(r)=2+2500*exp(−0.274*r)+ 100*exp(−0.022*r), (3)

where r is the instantaneous frequency.

### Excitatory mossy fiber connections

CN neurons are excited by mossy fibers [38]. The mossy fiber to CN neuron synapse is characterized by the presence of both AMPA and NMDA receptors [38]. NMDA receptors of this synapse are characterized by two components: a fast component (F_NMDA_) that shows weak voltage dependence and much slower component (S_NMDA_) that shows strong voltage dependence. NMDA voltage dependence is modeled [21] according to the equation:

$$f\left(V\right)=\;\frac{1}{\left(1+s1*\text{exp}\left(-s2*V_{m}\right)\right)}$$ (4)

AMPA, F_NMDA_, S_NMDA_ time constants and peak synaptic conductance values, parameters s1 and s2 of voltage dependence were obtained from [38] (S1 Table). We assumed a total of 100 mossy fiber synapses distributed randomly on the dendrites. Mossy fibers in the model were described by Poisson process with a mean firing frequency of 5 Hz, mimicking the background-firing rate of these fibers [39]. AMPA and NMDA synaptic kinetics were temperature corrected using experimentally determined Q_10_ values for synaptic current kinetics (Q_10_ = 2) [35,36].

### Generation of synthetic PN spikes

PN simple spike trains contain highly regular inter-spike intervals (ISIs) and hence cannot be described accurately by a Poisson process [12]. Shin *et al* [13] showed that regular patterns of PN simple spike ISIs can be described by higher order gamma processes while pause ISIs can be described by lower order gamma processes. Moreover, the estimated orders from regular patterns and pause ISIs followed gamma distributions indicating the presence of multiple processes underlying these two ISI types. Due to the limited availability of experimental spike trains, we amplified the data by generating numerous synthetic PN simple spike trains based on the properties of experimental ones.

Synthetic PN simple spikes were generated based on the method described in [12] using spike trains from experimental data [7]. Briefly summarized, synthetic spikes were generated in three steps after deleting the pauses in the simple spikes caused by complex spikes: for each experimental spike train, interspike intervals (ISIs) were categorized as regular patterns (group of regular ISIs) or pauses based on a measure of short-range variability (CV2) values (step 1, S5 Fig) [11,12,36]. For each of the regular patterns (step 2) or pauses (step 3) in the experimental spike train, a corresponding regular pattern or pause was generated for the synthetic spike train based on gamma distribution statistics. The similarity between experimental and synthetic ISIs was determined by Kolmogorov-Smirnov test. None of the generated synthetic ISIs was significantly different from experimental ones (99% confidence interval, p>0.01). S6 Fig compares the distribution of experimental and synthetic ISIs for 9 randomly selected data samples. The distribution of experimental and synthetic ISIs closely matched each other.

Pause synchrony is defined by either having the pause beginning or pause ending spike synchronized or no spikes (overlapping) synchronized across the population of PN inputs. We introduce synchrony by randomly selecting a pause that was longer than the threshold (either 20 or 40 ms) and temporally aligning it with pauses from other spike trains. The pause thresholds were selected so that they are greater than the spontaneous PN simple spike ISIs in *vivo* [12]. We used two different values of pause threshold so as to compare the effect of pause length on the spiking response of the CN neuron. As the exact amount of PN synchronization reaching a CN neuron is unknown (see Introduction and Discussion), we systematically increased the amount of synchronization in the presynaptic PN pauses and generated sets of spike trains in which 25, 50, 75 and 100% of the selected pauses were synchronized. Note that this way the ISIs before and after the pauses are still randomized in accordance to the real spike trains.

For simulations where we tested the role of PN firing rate before the pause on CN neuron’s firing rate post pause onset, average PN firing rate during each trial was forced to a particular frequency, ‘fPN'. We used the same number of trials for each fPN. Average PN firing rate for a trial was forced to fPN in the following way: For each PN participating in the synchronous pause, a pause exceeding a threshold of 20 ms and whose preceding spiking for a time period of 100 ms has a firing frequency of fPN, was randomly selected. We then temporally aligned both the pause and preceding spiking with their corresponding counterparts from other PN spike trains. For certain PNs where no desired fPN could be found before a pause, a pause exceeding the threshold was randomly selected.

### Data analysis

Neuronal spike times were recorded from the simulated CN neurons. The firing rate during synchronous pauses was computed and compared to a control condition (i.e., a model run with the same PN and mossy fiber spike sets, but without the synchronous pause) to investigate changes in the firing rate caused by the release of inhibition. We measured the spike latency with respect to the onset of synchronous pause, the standard deviation/median absolute deviation of which was then used to further assess the degree of time-locking.

To avoid the stochastic influences inherent to the synthetic spike trains, we performed 100 or more simulations for each input regime and computed the average change in firing rate, as well as the standard deviation/median absolute deviation of spike latency. We used standard deviation of latency to first spike within the synchronous pause if the distribution of spike latencies of both distributions followed the normal distribution. If either one of the distribution was not normal, the median absolute deviation was used. The normality of the distribution was established by Lilliefors test [40] using 99% confidence interval. A small standard deviation/median absolute deviation means the neuron is firing at almost the same time after a synchronous pause and hence greater accuracy in time-locking or spike timing precision to synchronous pause. We used bootstrapping and one-tailed t-tests to establish the significance and we assumed a confidence interval of 95%. Reliability of increase in firing rate was calculated as percentage of trials showing an increase in firing rate (Fig 3D). Population spike timing histogram (PSTH) of all PNs spikes projecting on to CN neuron were constructed by binning the spike times using 1ms bin size, and normalizing it to total number of spikes of the PSTH. The PSTH was constructed using datasets of the 100% PN synchronization (Figs 3, 4 and 8) condition to indicate the type of synchronization (pause beginning or ending or overlapping).

Vector strength [41,42] quantifying the degree of time-locking of spikes for various synchronization conditions was quantified in the following way: The spike latency during synchronous pause for each trial was represented as a vector on the unit circle by an angle θ where θ varies from 0 to 2π. Vector strength was computed by performing a vector addition of all vectors from various trials and was normalized to the number of trials. The outcome of this procedure produces a number on [0,1], where 0 means completely random spike times and 1 indicates perfect time-locking of all spikes. Significance of vector strength was established by the Rayleigh statistic [43]. The threshold for vector strength calculated by Rayleigh test is 0.2139. Rayleigh test's critical Z value for 100 trials and 99% confidence interval is 4.575.

For simulations where we tested the role of PN firing rate before the synchronous pause on the CN neuron’s firing rate following the immediate pause, the CN neuron’s firing rate was computed for a period of 1s from synchronous pause onset and compared to a control condition (model run without synchronized spike and pause sequence). Linear regression was used to fit a straight line through the data points. Correlation was measured with Pearson’s correlation as

$$r=\;\frac{cov\left(X,Y\right)}{\sigma_{X}\times \sigma_{Y}}$$ (5)

where *cov*(*X*, *Y*) is the covariance and σ the standard deviation.

## Supporting Information

S1 Fig CN neuron's firing rate increase mediated by pause ending synchronization and pause overlapping synchronization are not significantly different from each other. Analysis for 20 ms (A-D) and 40 ms (E-H) synchronous pauses. (A&E) Population spike timing histogram of all PNs projecting onto the CN neuron. (B&F) Increase in firing rate of CN neuron quantified for both pause ending and pause overlapping conditions and for low input gain. (C&G) Same for medium input gain. (D&H) Same for high input gain.
(TIF)

S2 Fig Effect of increasing PN pause synchronization on the rate increases of CN neuron for pause beginning type (A-D) and pause overlapping type (E-H) synchronization. (A&E) Population spike timing histogram of all Purkinje cells projecting onto CN neuron. Pause beginning type synchronization is illustrated in A and pause overlapping type is explained in E. (B&F) Increase in firing rate of the CN neuron during synchronous pause of length 20 ms and 40 ms and for low gain condition. (C&G) Same for medium gain condition. (D&H) Same for high gain condition. In all panels red color stands for 20 ms global pause period, blue for 40 ms global pause period. Error bars are represented by shaded region. (*) represents pairwise comparisons between results of different amount of input synchronization (25%-50%, 50%-75%, 75%-100%) that are significant (p<0.05) and (^) represents insignificant comparisons (p> = 0.05).
(TIF)

S3 Fig Time-locking of CN neuron spikes quantified for pause ending and pause overlapping synchronization condition. (A&E) Population spike timing histogram of all PNs projecting onto the CN neuron. Note the presence of synchronous pause of length 20 ms (A) and 40 ms (E) for both types of synchronization. (B&F) Variability of latency calculated from 100 trials for pause ending and pause overlapping type synchronization and low gain condition. (C&G) Same for medium gain condition. (D&H) Same for high gain condition. In all panels red color stands for pause ending condition, blue for pause overlapping condition. (*) represents comparisons of variability in latency between pause ending and pause overlapping type synchronization that are significant (p<0.05) and (^) represents insignificant comparisons (p> = 0.05).
(TIF)

S4 Fig Effect of pause length on the coding strategy of CN neuron. (A&E) Population spike timing histogram of all Purkinje cells projecting onto the nuclear neuron. Note the presence of synchronous pause beginning spikes and pause length of 20 ms (or 40 ms). Plots B,C and D represents quantification of variability in latency of CN neuron’s spiking for low (B), medium (C) and high input gain (D) condition respectively. Plots F,G and H represents quantification of increase in firing rate of the nuclear neuron for low gain (F), medium gain (G) and high gain (H) condition respectively. (*) represents appropriate comparisons that are significant (p<0.05) and (^) represents insignificant comparisons (p> = 0.05).
(TIF)

S5 Fig Generation of synthetic PN spikes. Synthetic PN spikes were generated using a three-step process: First we segregated the experimental ISIs into regular firing patterns (group of regular ISIs, blue) and pause ISIs (red) (step 1). For each regular firing pattern a corresponding synthetic regular firing pattern was generated based on gamma distribution statistics from [13] (step 2). A similar procedure was followed for pauses too where for each of the pauses in the experimental spike train a corresponding pause was generated based on gamma distribution statistics mentioned in [13] (step 3). RP-Regular pattern, P-Pauses.
(TIF)

S6 Fig Comparison of distribution of experimental and synthetic PN ISIs. A-I: Distribution of experimental and synthetic PN ISIs for nine randomly selected PNs. The similarity between experimental and synthetic ISIs was determined by Kolmogorov-Smirnov test. None of the generated synthetic ISIs was significantly different from experimental ones (99% confidence interval, p>0.01).
(TIF)

S1 Table Synaptic parameters used in the model and steady state release probability and time constants for synaptic depression. (DOCX)
