# Supplementary figures and images for: Cerebellar Nuclear Neurons Use Time and Rate Coding to Transmit Purkinje Neuron Pauses
Source: PLoS Comput Biol. 2015 Dec 2;11(12):e1004641. doi: 10.1371/journal.pcbi.1004641 (PMC4668013; doi:10.1371/journal.pcbi.1004641)

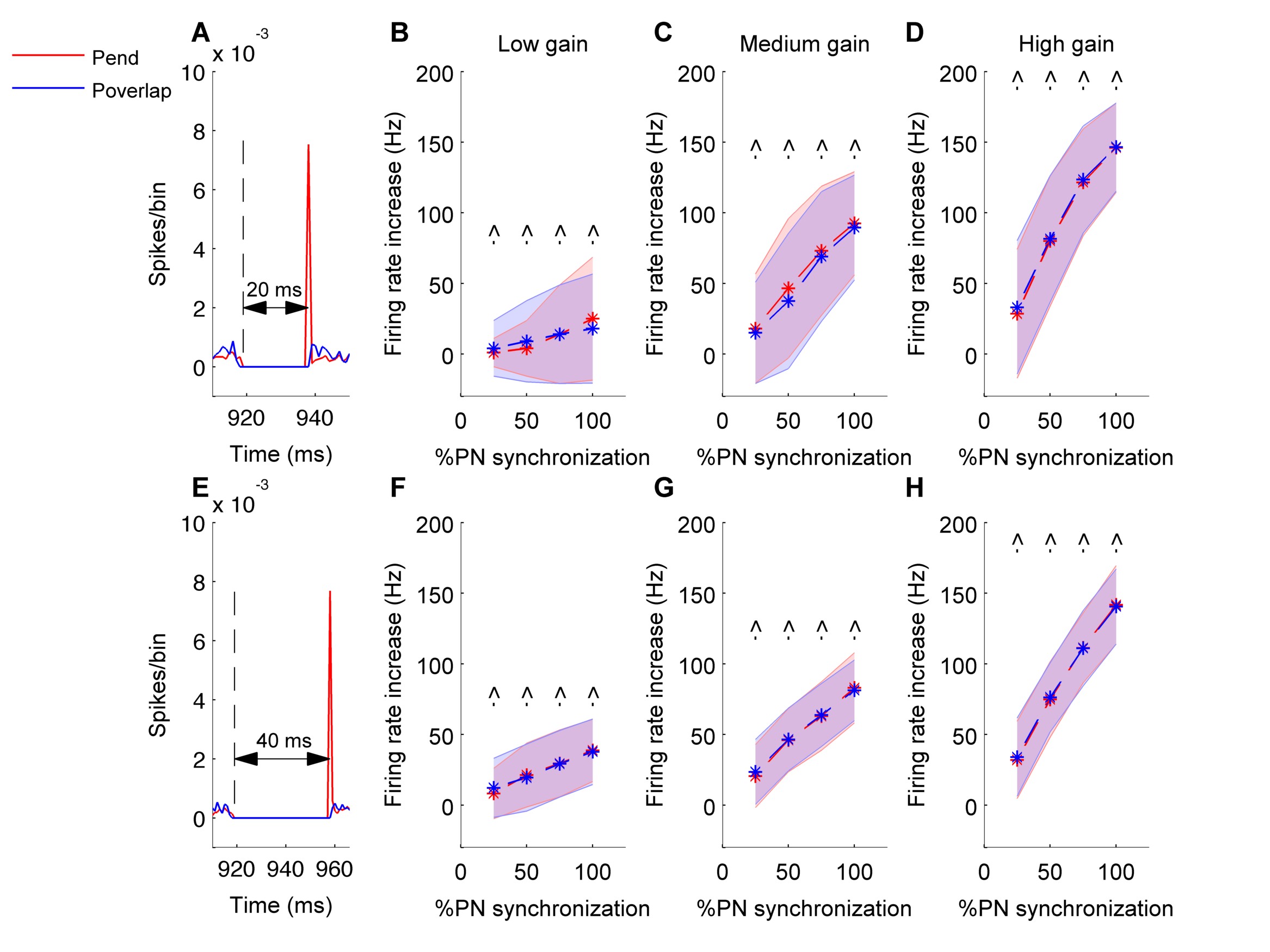

Supplement: S1 Fig — Analysis for 20 ms (A-D) and 40 ms (E-H) synchronous pauses. (A&E) Population spike timing histogram of all PNs projecting onto the CN neuron. (B&F) Increase in firing rate of CN neuron quantified for both pause ending and pause overlapping conditions and for low input gain. (C&G) Same for medium input gain. (D&H) Same for high input gain. (TIF) [file pcbi.1004641.s001.tif]

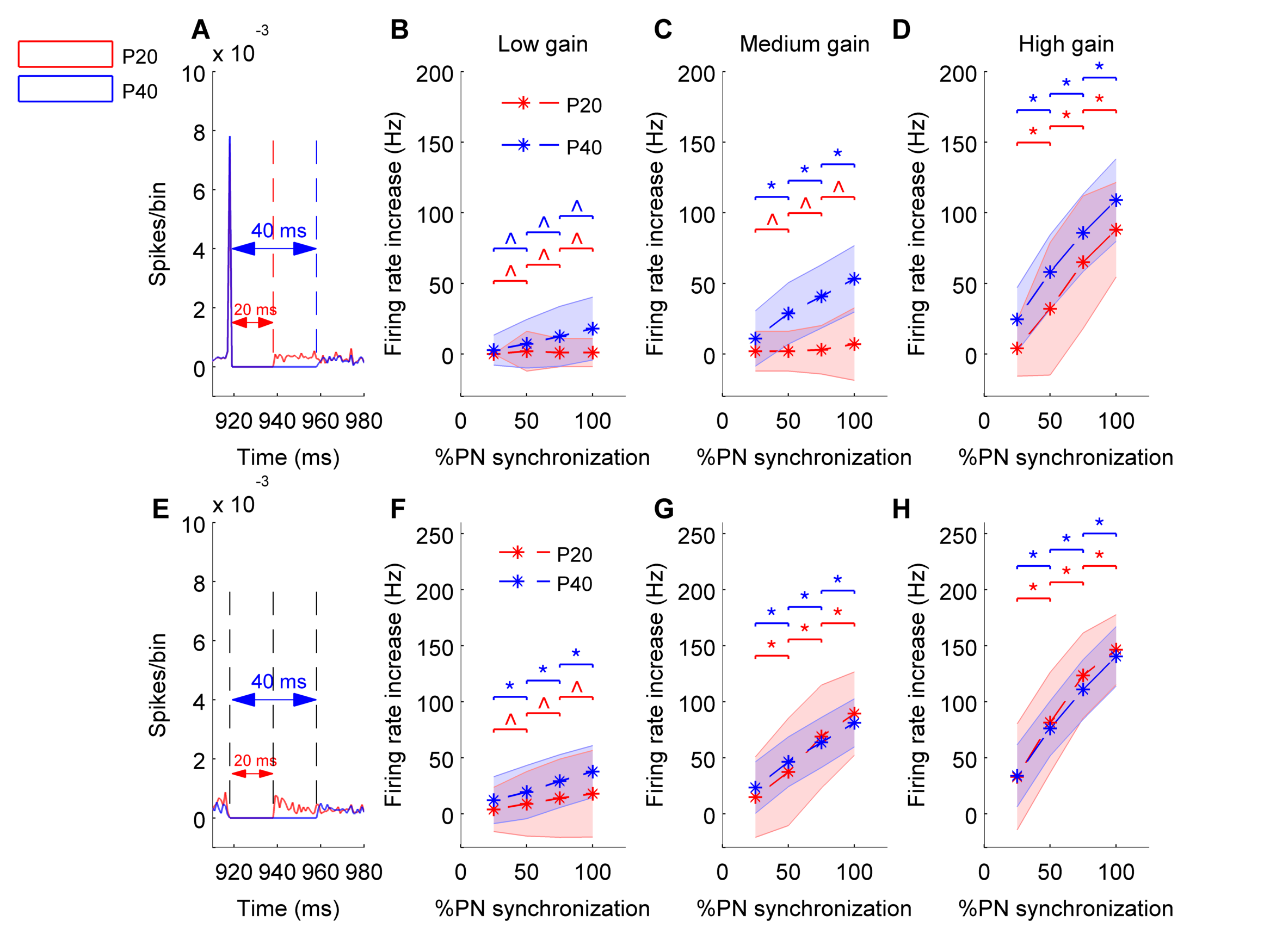

Supplement: S2 Fig — (A&E) Population spike timing histogram of all Purkinje cells projecting onto CN neuron. Pause beginning type synchronization is illustrated in A and pause overlapping type is explained in E. (B&F) Increase in firing rate of the CN neuron during synchronous pause of length 20 ms and 40 ms and for low gain condition. (C&G) Same for medium gain condition. (D&H) Same for high gain condition. In all panels red color stands for 20 ms global pause period, blue for 40 ms global pause period. Error bars are represented by shaded region. (*) represents pairwise comparisons between results of different amount of input synchronization (25%-50%, 50%-75%, 75%-100%) that are significant (p<0.05) and (^) represents insignificant comparisons (p> = 0.05). (TIF) [file pcbi.1004641.s002.tif]

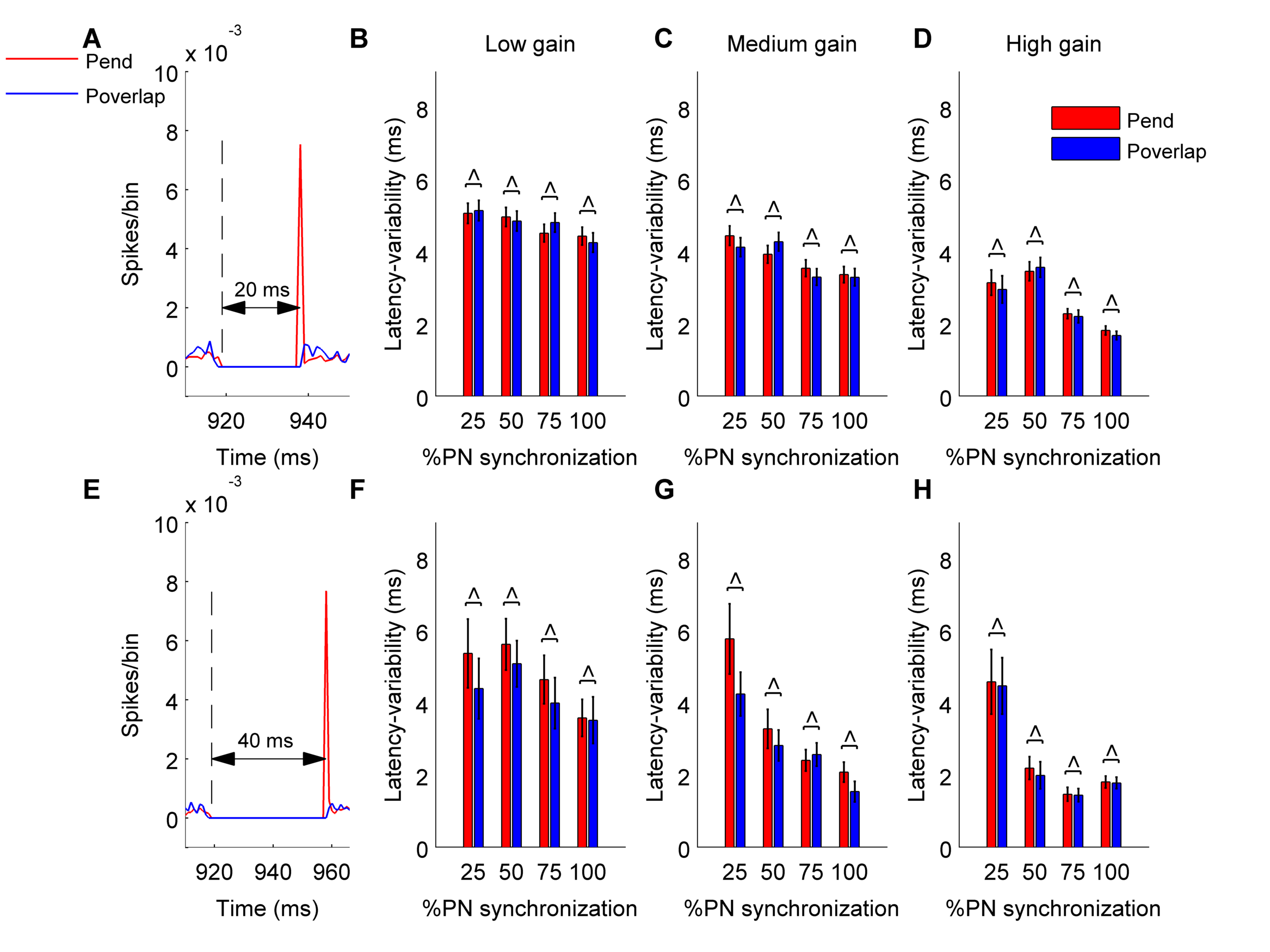

Supplement: S3 Fig — (A&E) Population spike timing histogram of all PNs projecting onto the CN neuron. Note the presence of synchronous pause of length 20 ms (A) and 40 ms (E) for both types of synchronization. (B&F) Variability of latency calculated from 100 trials for pause ending and pause overlapping type synchronization and low gain condition. (C&G) Same for medium gain condition. (D&H) Same for high gain condition. In all panels red color stands for pause ending condition, blue for pause overlapping condition. (*) represents comparisons of variability in latency between pause ending and pause overlapping type synchronization that are significant (p<0.05) and (^) represents insignificant comparisons (p> = 0.05). (TIF) [file pcbi.1004641.s003.tif]

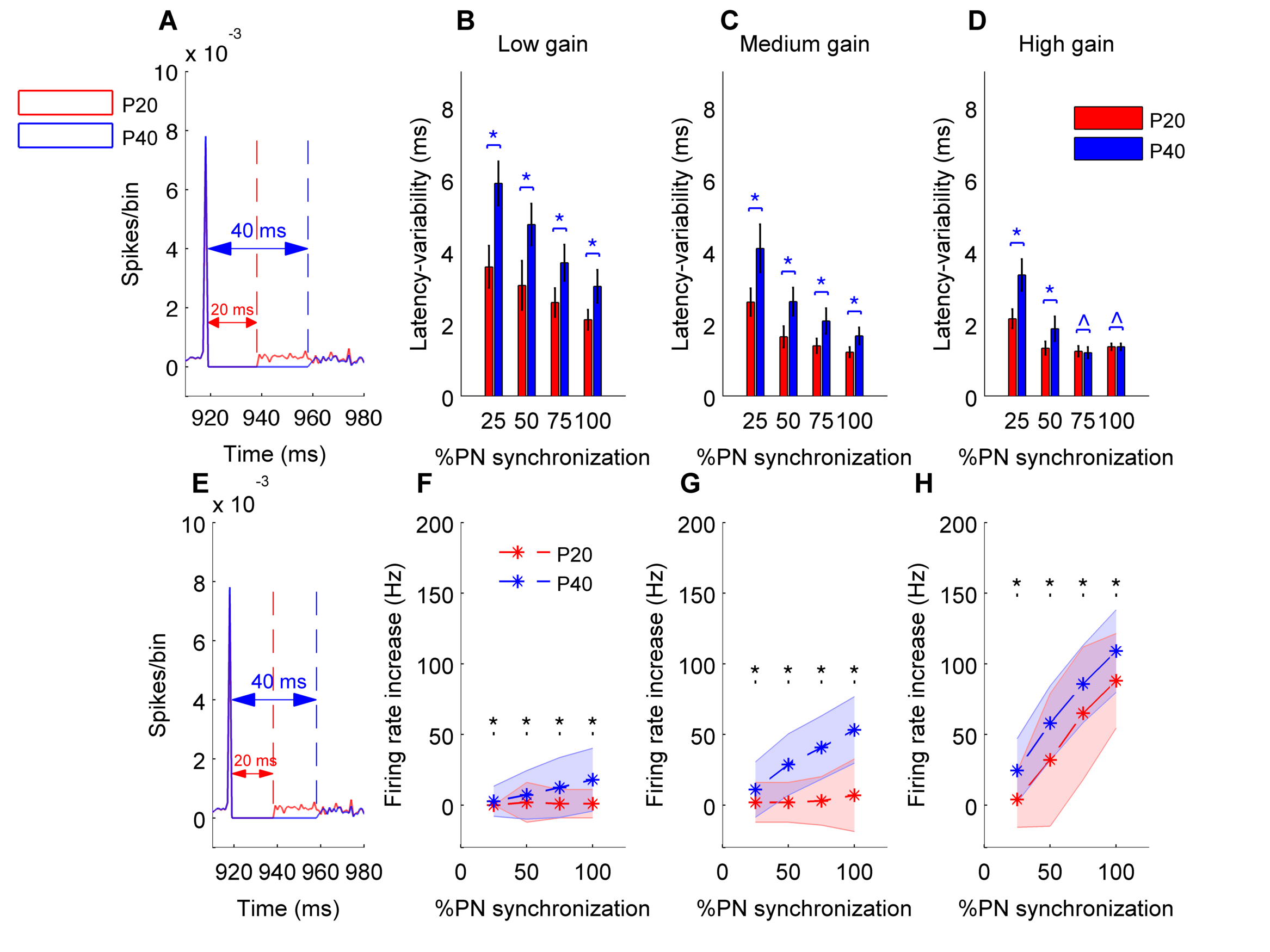

Supplement: S4 Fig — (A&E) Population spike timing histogram of all Purkinje cells projecting onto the nuclear neuron. Note the presence of synchronous pause beginning spikes and pause length of 20 ms (or 40 ms). Plots B,C and D represents quantification of variability in latency of CN neuron’s spiking for low (B), medium (C) and high input gain (D) condition respectively. Plots F,G and H represents quantification of increase in firing rate of the nuclear neuron for low gain (F), medium gain (G) and high gain (H) condition respectively. (*) represents appropriate comparisons that are significant (p<0.05) and (^) represents insignificant comparisons (p> = 0.05). (TIF) [file pcbi.1004641.s004.tif]

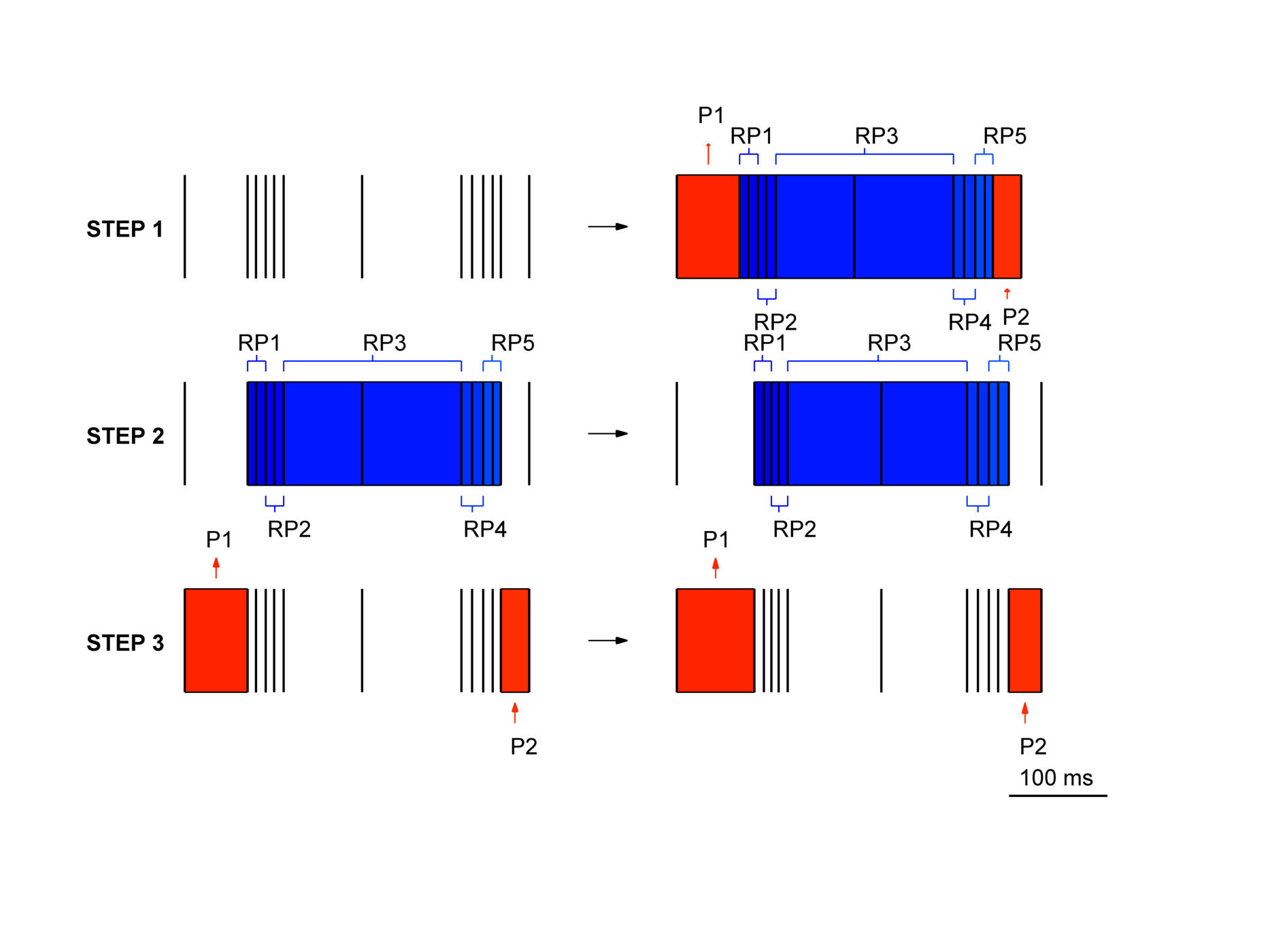

Supplement: S5 Fig — Synthetic PN spikes were generated using a three-step process: First we segregated the experimental ISIs into regular firing patterns (group of regular ISIs, blue) and pause ISIs (red) (step 1). For each regular firing pattern a corresponding synthetic regular firing pattern was generated based on gamma distribution statistics from [13] (step 2). A similar procedure was followed for pauses too where for each of the pauses in the experimental spike train a corresponding pause was generated based on gamma distribution statistics mentioned in [13] (step 3). RP-Regular pattern, P-Pauses. (TIF) [file pcbi.1004641.s005.tif]

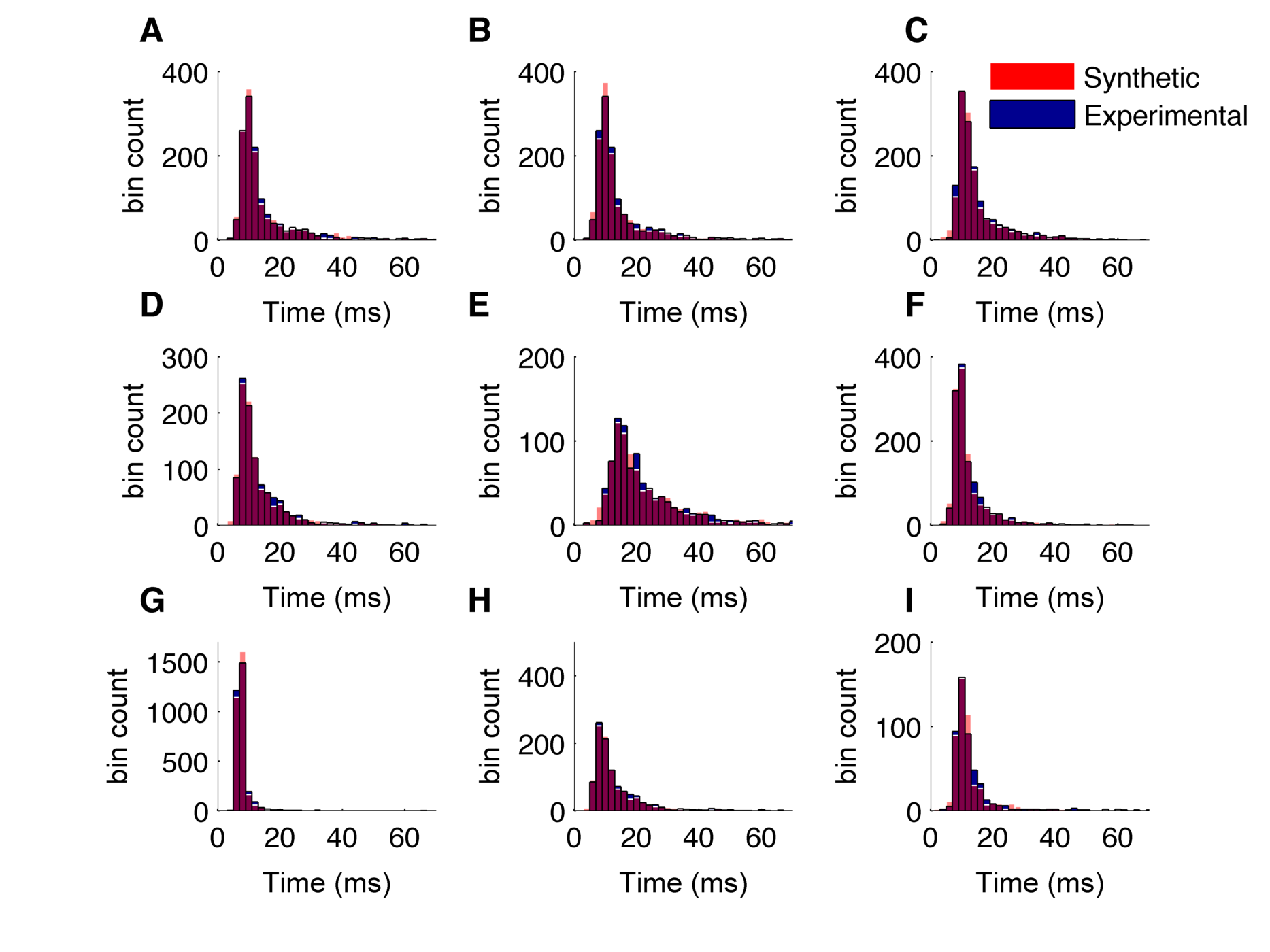

Supplement: S6 Fig — A-I: Distribution of experimental and synthetic PN ISIs for nine randomly selected PNs. The similarity between experimental and synthetic ISIs was determined by Kolmogorov-Smirnov test. None of the generated synthetic ISIs was significantly different from experimental ones (99% confidence interval, p>0.01). (TIF) [file pcbi.1004641.s006.tif]
