# Supplementary material for: Cerebellar Nuclear Neurons Use Time and Rate Coding to Transmit Purkinje Neuron Pauses
Source: PLoS Comput Biol. 2015 Dec 2;11(12):e1004641. doi: 10.1371/journal.pcbi.1004641 (PMC4668013; doi:10.1371/journal.pcbi.1004641)
Supplement: S1 Table — (DOCX) [file pcbi.1004641.s007.docx]

| **Parameter name** | **Value** |
| --- | --- |
| GABA- τ_rise_, τ_decay, gmax_ | 0.1 ms, 2.4 ms and 11.7 nS respectively |
| AMPA- τ_rise_, τ_decay, gmax_ | 0.5 ms, 7.1 ms and 3.25 nS respectively |
| F_NMDA_- τ_rise_, τ_decay, gmax_ | 5 ms, 20.2 ms and 6 nS respectively |
| S_NMDA_- τ_rise_, τ_decay, gmax_ | 5 ms, 136.4 ms and 6 nS respectively |
| F_NMDA_-s1, s2 | 0.002 mM, 0.109 respectively |
| S_NMDA_-s1, s2 | 0.25 mM, 0.057 respectively |
| E_rev (excitatory)_, E_rev (inhibitory)_ | 0 mV and -75 mV respectively |
| Q10 (synaptic kinetics) | 2 |

| **Instantaneous frequency**  (Hz) | **Steady state release probability** **(R_ss_)** | **Time constant** **(τ )**  (ms) |
| --- | --- | --- |
| 1 | 0.43 | 2001 |
| 10 | 0.34 | 243.6 |
| 20 | 0.29 | 76.82 |
| 30 | 0.25 | 54.35 |
| 40 | 0.22 | 43.5 |
| 50 | 0.19 | 35.3 |
| 60 | 0.17 | 28.7 |
| 70 | 0.15 | 23.4 |
| 80 | 0.14 | 19.2 |
| 90 | 0.13 | 15.8 |
| 100 | 0.12 | 13.08 |
| 150 | 0.09 | 5.6 |
| 200 | 0.08 | 3.2 |
